# Sensory Stream Adaptation in Chaotic Networks

**DOI:** 10.1038/s41598-017-16478-z

**Published:** 2017-12-04

**Authors:** Adam Ponzi

**Affiliations:** 1 IBM T.J. Watson Research Center, Yorktown Heights, NY USA; 2 Okinawa Institute of Science and Technology Graduate University (OIST), Okinawa, Japan

## Abstract

Implicit expectations induced by predictable stimuli sequences affect neuronal response to upcoming stimuli at both single cell and neural population levels. Temporally regular sensory streams also phase entrain ongoing low frequency brain oscillations but how and why this happens is unknown. Here we investigate how random recurrent neural networks without plasticity respond to stimuli streams containing oddballs. We found the neuronal correlates of sensory stream adaptation emerge if networks generate chaotic oscillations which can be phase entrained by stimulus streams. The resultant activity patterns are close to critical and support history dependent response on long timescales. Because critical network entrainment is a slow process stimulus response adapts gradually over multiple repetitions. Repeated stimuli generate suppressed responses but oddball responses are large and distinct. Oscillatory mismatch responses persist in population activity for long periods after stimulus offset while individual cell mismatch responses are strongly phasic. These effects are weakened in temporally irregular sensory streams. Thus we show that network phase entrainment provides a biologically plausible mechanism for neural oddball detection. Our results do not depend on specific network characteristics, are consistent with experimental studies and may be relevant for multiple pathologies demonstrating altered mismatch processing such as schizophrenia and depression.

## Introduction

In predictably structured sensory streams like music, speech and visual input during motion the brain doesn’t simply respond to external events but anticipates them^1–4^. Studies of temporal expectation, also known as implicit timing^5^, demonstrate the importance of predictability of when stimuli will occur. Task performance is enhanced if stimuli occur at expected times according to an established rhythm^6–8^. The sensory streams used are also found to phase entrain low-frequency brain oscillations^9–12^. Phase entrainment increases with the temporal regularity of the sensory stream and correlates with behavioural performance. It is not limited to sensory cortices^13^; activity patterns in larger scale cortical^14,15^ and subcortical networks^16^ are also modulated by temporal expectation.

Related studies show that implicit expectations of upcoming stimulus itself can also strongly affect neural response. Stimulus specific adapatation (SSA) occurs in single cells^17^ and in multi-unit activity^18^. Here the response to a stimulus is in general suppressed after multiple presentations while responses to oddball stimuli may be enhanced^19^. SSA occurs in both excitatory and inhibitory cells^20,21^ and in multiple areas from midbrain to cortex^22^. Type expectation also occurs at the neuronal population level as oscillatory mismatch responses (MMR) after stimulus offset in EEG/MEG^23–27^, ECoG^19^, and LFP^28^. Most importantly adaptation can occur to whole stimulus sequences on multiple time scales simultaneously^18,29^ while the effects are stronger when streaming is temporally regular^30^. Mismatch responses are altered in pathologies, notably schizophrenia, traumatic brain injury and Alzheimers disease, and can be used as diagnostic tools^31–35^.

Whether population and single cell modulations originate from the same underlying mechanism is controversial^36–39^. SSA is thought to depend on single cell adaptation but studies have also implicated network mechanisms^21,40,41^ including recurrent inhibition^42,43^. MMR has been suggested to involve synaptic adaptation^44^ as well as memory^45^ and predictive coding^46^ based mechanisms. Intriguing recent investigations have highlighted a possible interaction of SSA and MMR through recurrent network inhibition^20,47^.

Neural information processing may be embedded in recurrent network dynamics^48–53^ which are also thought to underlie ongoing brain oscillations^54–56^. Recent modeling work^57^ has demonstrated entrainment of low frequency stable network oscillations by transcranial magnetic stimulation. However low frequency brain oscillations may also be chaotic^58–62^. We find that the neuronal correlates of sensory stream adaptation emerge when unstructured recurrent networks generate fairly low dimensional chaotic dynamics. We show that this is because entrainment of chaotic oscillations^63^ results in the complex close to critical dynamics^64,65^ which supports the history dependent response on long behaviourally relevant timescales necessary for sensory stream adaptation^66,67^.

## Results

Sensory stream adaptation is a general phenonemon observed across multiple brain regions on multiple timescales. Therefore to demonstrate the hypothesis in the simplest way possible we use numerical simulations of a very standard random network of firing rate units without any synaptic plasticity or facilitation mechanisms (see Methods.) We are primarily interested in low frequency oscillations 1 ~ 10 Hz in population measures such as LFP and EEG. We therefore use a model derived from neurotransmitter fluctuations which average over spikes. The model of low frequency oscillations used in^57^ also employs adapting membrane potentials. Although highly simplified, rate networks can provide useful insight into elementary principles of real brain network dynamics^60,68,69,78–80^ where an individual rate unit reflects the activity of a local circuit including multiple cells of several types when spiking is irregular and asynchronous^80,81^.

**Figure 1:**
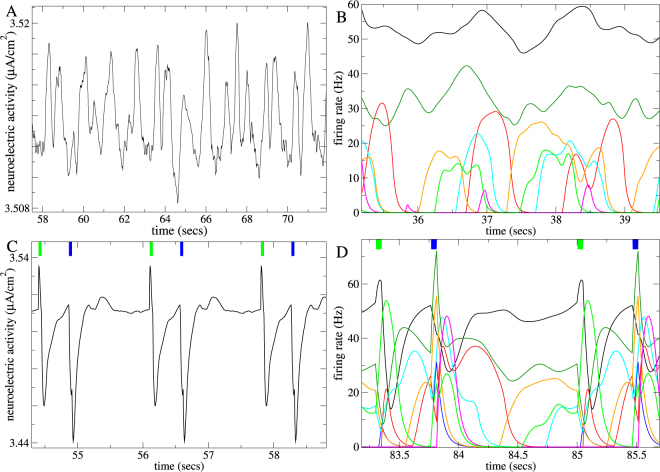
Illustration of chaos stabilization by sensory streaming in a particular inhibitory network simulation. (**A**,**C**) Mean neuroelectric activity averaged across all 500 units in (**A**) the autonomous network simulation when continuously driven by the background input *X*, and (**C**) the network driven by the *AB* sensory stream where the ITI following *A* is always 415 ms and the ITI following *B* is always 1185 ms. (**B**,**D**) Firing rate for several individual units from (**B**) the autonomous simulation in (**A**), and (**D**) the sensory stream driven simulation in (**C**). (**C**,**D**) Ticks indicate the 50 ms sensory stimulus presentations, *A* (green) and *B* (blue) in the sensory stream. During ITI periods between stimulus presentations the network is driven by the same background input *X* as in (**A**,**B**). (**A**–**D**) All simulations are deterministic.

The activity of each unit in the network depends on its net synaptic input transformed by the transfer function of a type I cell. The precise values of the model parameters, as well as the form of the transfer function itself, are not crucial. The important point is that the network should generate fairly low dimensional chaotic dynamics. Both purely inhibitory and excitatory-inhibitory rate networks^60,68^ show stable and chaotic dynamical regimes as network parameters, for example the connection probability, are varied. Such weakly chaotic dynamics occurs in fairly sparsely connected networks, (connection probability ~0.15) and in the close to balanced but inhibition dominated regime in excitatory-inhibitory networks. Despite the absence of timescale parameters exceeding 50 ms such networks can generate large slow coherent oscillatory population fluctuations (Fig. 1(A,B)) on the extended behaviourally relevant timescales used in the sensory streaming tasks we investigate here.

Here we focus mainly on inhibitory networks however the capacity for more general larger excitatory-inhibitory networks to show similar behaviour is also demonstrated. Indeed several recent studies have highlighted network inhibition as crucial in deviance detection and its interaction with slow oscillations^20,21,47^. Inhibition is important in sensory network dynamics^82^ and may modulate the interaction of internal dynamics and sensory input by controlling network stability^59^. As a simple proxy for the LFP/EEG we use the total absolute size of the average input current to a rate unit^57,70,83,84^, which we term the ‘neuroelectric activity’^57^.

The sensory streams we investigate are made up of several stimuli applied in sequence. A stimulus represents a static visual noise patch or an auditory stimulus composed of random frequencies, for example. Each stimulus is modeled as a fixed set of excitatory driving inputs (see Methods) one of which is applied to each of the units in the network for the duration of the stimulus. The driving inputs which make up any given stimulus are random, drawn independently from a single given distribution once and then fixed for the duration of the simulation. Therefore all stimuli are identical statistically (see Methods).

Most of the sensory streams we investigate include only two sensory stimuli, denoted *A* and *B*. They are always applied to the network for brief 50 ms periods, as is common in sensory streaming experiments. During inter-trial-intervals (ITIs) between stimuli the network is driven by a different input we denote ‘background’ input *X*. Input *X* can represent external sensory input during ITI periods or alternatively purely internally generated excitatory driving or a combination of the two. In order to demonstrate that our results do not require that sensory stimuli provide stronger levels of excitation than the background input we also draw input *X* from the same distribution as the sensory stimuli *A* and *B*. The ITIs in a sensory stream can be of fixed or variable length but the mean length investigated is usually 800 ms. This generates sensory streams with stimulus presentation frequencies typically used in experiments.

Simulations (except those for demonstration of dynamics and for calculations of Lyapunov exponents (see below)) include noise. The noise is not necessary for the results here. It has been included to show that our results are resilient to its presence (see Methods). In the first part of this paper we illustrate how chaos entrainment works to generate reliable, distinct and history dependent stimulus response in this model. In the second part we show how these properties can explain several experimental results in sensory stream adaptation.

### Sensory streams entrain chaotic network activity

First we illustrate how sensory streams entrain chaos in a particular exemplar 500 unit inhibitory network simulation. Figure 1(A,B) show the spontaneous activity generated by the network when it is constantly driven by the background input *X*. (In the following we refer to the activity endogenously generated by the network under the constant driving of the background input *X* as the ‘autonomous’ activity.) The random looking oscillations in the mean neuroelectric activity (Fig. 1(A)) and in the individual units activity (Fig. 1(B)) suggest that the autonomous activity is chaotic.

**Figure 2:**
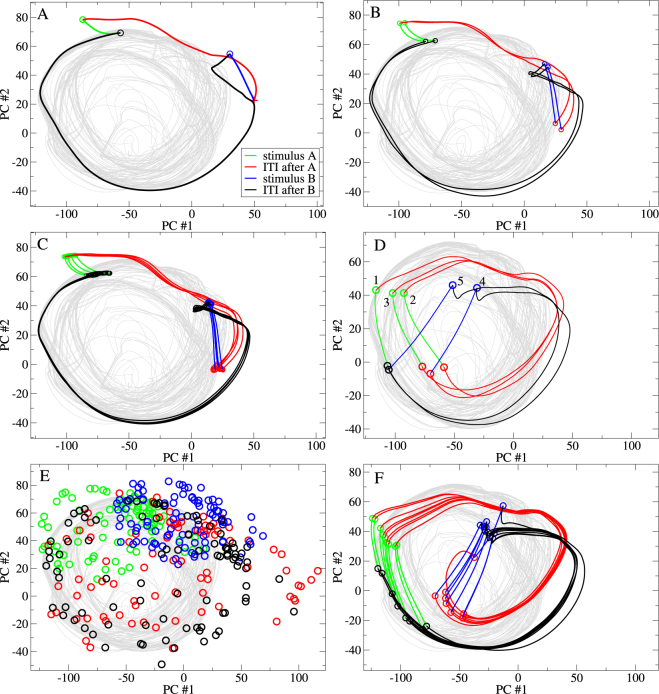
Comparison of principal component projections for the network simulation studied in Fig. 1 under different sensory streams composed of two 50 ms stimuli, *A* and *B*. (**A**) *A* and *B* alternate, ITI following *A* 415 ms. ITI following *B* 1185 ms. (**B**) *A* and *B* alternate, ITI following *A* 500 ms. ITI following *B* 1100 ms. (**C**) *A* and *B* alternate, ITI following *A* 521 ms. ITI following *B* 1079 ms. (**D**) *AAABB* sensory stream with all ITIs fixed at 800 ms. (**E**) *A* and *B* sequence is scrambled. ITIs are drawn from an exponential distribution with mean 800 ms. (**F**) *A* and *B* alternate. ITIs are composed of a fixed 700 ms plus an exponentially distributed period of mean 100 ms. (**A**–**F**) Coloured line segments show the trajectory of the sensory stream driven activity projected onto the first two principal components of the autonomous activity. Segments are coloured according to the epoch within the sensory stream. Lines show trajectory segments, symbols show trajectory segment endpoints (see key.) In (**C**) for clarity only four trajectory segments are shown, although all trajectory segment endpoints are shown. In (**D**) stimulus epoch endpoints are labelled by their order in the *AAABB* stream. (**E**) Only segment endpoints are shown. (**A**–**E**) Grey line shows the trajectory of the first two principal components of the autonomous activity. All simulations are deterministic of length 150 secs. Driven Lyapunov exponents *λ*_*D*_ are (**A**) −0.001, (**B**) −0.0001 (**C**) 0.0002, (**D**) −0.001, (**E**) −0.0006 (**F**) −0.0003.

We use principal component analysis to visualize the network chaotic attractor. The grey line in Fig. 2(A) shows the trajectory of the autonomous activity projected in the space of its first two principal components. One particular trajectory is shown. However there are multiple other trajectories winding around the attractor, tangled up with the one shown. Which trajectory the system follows depends on the choice of initial condition. Each trajectory gradually fills the attractor as time progresses but trajectories also diverge from each other on shorter timescales. Due to this mixing property initially nearby points eventually become spread across the whole attractor.

This trajectory divergence is quantified by the maximal Lyapunov exponent. It measures the rate at which nearby dynamical trajectories diverge or converge. When the maximal Lyapunov exponent is positive network activity is chaotic and unstable to small perturbations, like noise, which can knock the system from one trajectory onto another. On the other hand in networks with stable dynamical attractors, like limit cycles and fixed points, small perturbations to trajectories decay and the maximal Lyapunov exponent is negative or zero. In this network simulation example (Figs 1(A) and 2(A, grey line)) the maximal Lyapunov exponent of the autonomous activity, denoted *λ*_*U*_, (see Methods) is positive, *λ*_*U*_ = 0.0012, confirming the presence of chaos.

Figure 1(C,D) shows what happens when the same network simulation is driven by a particular sensory stream, denoted the *AB* sensory stream. Here the two 50 ms stimuli, *A* and *B*, are presented in alternating sequence. The ITI following stimulus *A* is always 415 ms while that after *B* is always 1185 ms. Evidently the irregular variations in activity in the autonomous network simulation (Fig. 1(A,B)) become locked to the sensory stream in the driven simulation. The solid lines in Fig. 2(A) show the sensory stream driven trajectory projected onto the principal components of the autonomous activity. For visualization the trajectory has been divided into different sections coloured according to the epoch in the sensory stream. The trajectory appears to circulate in the vicinity of the autonomous attractor and is periodic with period of the sensory driving (here and in the following we use the terminology ‘periodic sensory stream’ to denote a sensory stream which repeats exactly at fixed time intervals). This driven network trajectory is fully stable as confirmed by calculation of the driven maximal Lyapunov exponent, denoted *λ*_*D*_ (see Methods) which is negative, *λ*_*D*_ = −0.0013, for this simulation in this particular sensory stream. This example therefore demonstrates the complete stabilization of autonomous rate chaos by the sensory stream.

Entrainment of chaos can also be partial. Figure 2(B,C) show examples from the same network simulation under the alternating *AB* sensory stream with the same stimuli *A* and *B* except that the ITI lengths have been changed slightly, while the mean ITI remains fixed at 800 ms. In Fig. 2(B) the driven trajectory is again fully stable, *λ*_*D*_ < 0, but it is now ‘mode locked’ with double the period of the driving, a behaviour also shown by stable network oscillators^57^. In Fig. 2(C) chaos is only *partially stabilized*, *λ*_*U*_ > *λ*_*D*_ > 0. Although the driven trajectory never quite repeats it is strongly restricted within a ‘bundle’ of much lower dimension than the chaotic attractor (grey). This is a general characteristic of chaotic synchronization^75,76,85–87^ and, if the sensory stream is periodic, phase synchronization^63^. Here phase synchronization is evident because any point in the stream occurs roughly at the same fixed phase around the trajectory on each cycle.

### Stabilized chaotic trajectories are strongly history dependent

In sensory stream adaptation experiments repeat presentations of a given stimulus must generate different distinct responses which are however reliable when the stream itself is repeated^51,52,74,77,88–91^. Here we illustrate why (fully and partially) stabilized chaos provides the ideal substrate for this^92,93^. Figure 2(D) shows the trajectory of the same network driven by the same stimuli *A* and *B* but now composed in a different sensory stream where the sequence *AAABB* is presented repeatedly with fixed 800 ms ITIs between all stimulus presentations. The trajectory is again stable, *λ*_*D*_ < 0, but stimulus responses are totally different to those under the *AB* stream, Fig. 2(A). The response to any given stimulus is strongly history dependent and adapted to the periodic sensory stream. Three distinct stimulus *A* responses and two distinct stimulus *B* responses can be seen. Such history dependent neural response is known in various task settings and brain regions. ^94^provides a recent example.

The distinctiveness of stimulus responses depends on how chaotic the autonomous network dynamics is, given by *λ*_*U*_. Points on trajectories which are preceded by more similar sensory streams tend to be closer in the state space. This is visible in Fig. 2(D) where the stimulus response endpoints (green circles) for the second and third presentations of *A* are closer together than they are to the first stimulus *A* endpoint. However as the strength and dimension of autonomous chaos increases ITI period trajectories become more divergent and the driven trajectory becomes spread over a larger region. Therefore the responses generated by individual stimulus presentations reflect longer and longer sequences of preceding stimuli.

On the other hand the reliability of a stimulus response depends on two factors. First, if chaos is not completely suppressed, Fig. 2(C), or if it is mode-locked, Fig. 2(B), repeat presentations of the same stimulus can generate slightly different responses, even though the dynamics is completely deterministic. This type of reliability is described by *λ*_*D*_ and in general decreases as it increases. It is evident from these examples that the effects of unsupressed chaos on response reliability can be very weak. This is because the ‘widths’ of the trajectory ‘bundles’ are small compared to the total variation around the trajectory phase. In fact partially stabilized chaos is particularly relevant for sensory stream adaptation since it combines fairly high reliability with high distinctiveness (as illustrated below.) A much stronger effect on stimulus response reliability originates directly from the sensory stream itself. In fully and partially stabilized chaotic networks, *λ*_*D*_ < *λ*_*U*_, response reliabilities for given stimuli decrease as the multiplicity of sensory streams which precede individual presentations of that stimulus increases. For example in the *AAABB* sensory stream, Fig. 2(D), stimuli of type *A* generate much less reliable responses than stimuli of type *B*.

### Chaos stabilization only weakly depends on stream predictability

Since stabilized chaos facilitates sensory stream adapted response it is important to investigate how chaos stabilization depends on network properties as well as on the properties of the sensory stream.

**Figure 3:**
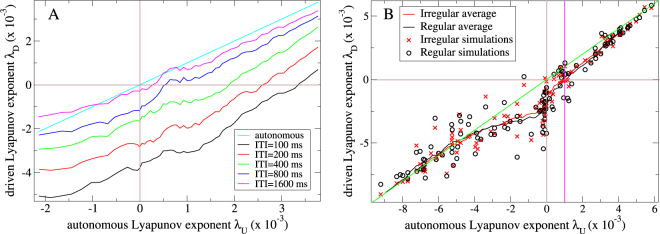
Maximal Lyapunov exponent analysis of stabilization of chaos by sensory streaming. Driven maximal Lyapunov exponent, *λ*_*D*_, versus the autonomous maximal Lyapunov exponent, *λ*_*U*_ for multiple different network simulations under (**A**) a temporally regular sensory stream where stimulus type, *A* or *B*, alternates and all ITIs are fixed and equal, for streams of different ITI lengths (see key, lines show 7 point moving averages each over 71 network simulations) and (**B**) the temporally regular sensory stream with 800 ms ITI and a fully irregular sensory stream where the stimulus type sequence is scrambled and ITIs are drawn independently from a exponential distribution with expectation 800 ms (see key, lines show 20 point moving averages). Green line indicates *λ*_*D*_ = *λ*_*U*_. Chaos is partially stabilized for simulations in the upper right quadrant below the green line, since here *λ*_*U*_ > *λ*_*D*_ > 0. Chaos is fully stabilized in simulations in the lower right quadrant since *λ*_*U*_ > 0 > *λ*_*D*_. Pink vertical line where the moving averages cross *λ*_*D*_ = 0 indicates the onset of full stabilization of chaos on average across multiple simulations. (**A**,**B**) All simulations are deterministic.

In Fig. 3 driven Lyapunov exponents *λ*_*D*_ are plotted versus autonomous Lyapunov exponents *λ*_*U*_ for many inhibitory network simulations generated with various network connection probabilities (see Methods.) Fig. 3(A) shows *λ*_*D*_ for streams where stimuli *A* and *B* are presented in alternating sequence separated by fixed and equal ITIs, for various ITI lengths. Chaotic network activity is generally stabilized somewhat by the sensory driving since *λ*_*D*_ < *λ*_*U*_ when *λ*_*U*_ > 0. The magnitude of stabilization, *λ*_*U*_ − *λ*_*D*_, is largest for networks with *λ*_*U*_ >≈ 0 and decreases to zero as networks become more strongly chaotic. The higher the stimulus presentation frequency the greater the stabilization, as also found in^95,96^. We find some stabilization occurs for ITI periods as short as 100 ms and as long as several seconds. These are natural timescales often employed in sensory streaming studies^6,9,10,18,19,25,98–101^.

Figure 3(B) compares *λ*_*D*_ for the alternating *AB* sensory stream with fixed 800 ms ITI and a maximally irregular sensory stream where ITI intervals are exponentially distributed with mean 800 ms and where stimuli *A* and *B* are applied in scrambled sequence. Although for any particular simulation *λ*_*D*_ can depend on the stream (as demonstrated by the example in Fig. 2), on average both periodic and random streams stabilize chaos roughly equally (Fig. 3(B) lines), (although there is a weak effect of randomness). The somewhat surprising finding that even completely random streams suppress chaos as much as periodic ones is important because it means stimuli in random streams also have the capacity to show stream history dependent response. If this were not the case stimulus response would become fully unreliable suddenly at some level of stream randomness. The point is illustrated in Fig. 2(E,F) where the trajectories under the maximally irregular and a weakly irregular sensory stream are compared. Stimulus responses and ITI period trajectories are much more reliable when activity is less irregular. Since both trajectories are almost equally stable relative response reliabilities are determined by the regularity of the sensory stream.

There have been multiple investigations of chaos control in neural networks, for example^53,77,92,95,97,102^. Here we don’t address it in detail. However simulations show large negative deflections in *λ*_*D*_ at stimulus onset and offset (see Supplemental Fig. S8 and Supplemental Text). This suggests that the strong dependence of stabilization on stimulus presentation frequency and weak dependence on randomness may be very roughly explained by a simple mechanism. When the autonomous Lyapunov exponent, *λ*_*U*_, generated when the network is driven by the background input *X,* is small there are only a few unstable directions which are confined within a low dimensional attractor manifold within the much higher dimensional space. During stimulus presentations the input *X* generated attractor is replaced by a different stimulus dependent one. This causes the activity state to move away from the *X* attractor region towards the new attractor region and then rapidly return after stimulus offset. Both these transient attractor switching pieces of the driven trajectory are stable. If the stabilizing effect of these kicks is sufficient to overcome the divergence on the *X* attractor then the driven trajectory will become stable overall, regardless of the temporal regularity or type of sensory stimulus presentations.

In the following we denote the approximate regime where chaos is fully or partially stabilized, 0 < *λ*_*U*_ < 0.003, for sensory streams with mean ITI around 800 ms, typically used in experiments the ‘suppressed chaos regime’. Here we focus on the role of network spontaneous activity in sensory stream adaptation, rather than how this activity arises from network structure. However for completeness in Supplemental Fig. S1 we investigate this regime in more detail. It is quite broad in biologically realistic regimes of network parameter space, spanning recurrent connection probabilities from about 0.1 to 0.25, while networks including excitatory units are inhibition dominated. The point where chaos is just suppressed in fully inhibitory networks has connection probability around 0.17 and mean synpatic conductance around 0.47 *nS*/*cm* ^2^ when average firing rates are 1 Hz (see Methods and Supplemental Methods.)

### Reliable and distinct deviancy level dependent response in roving sensory streams

**Figure 4:**
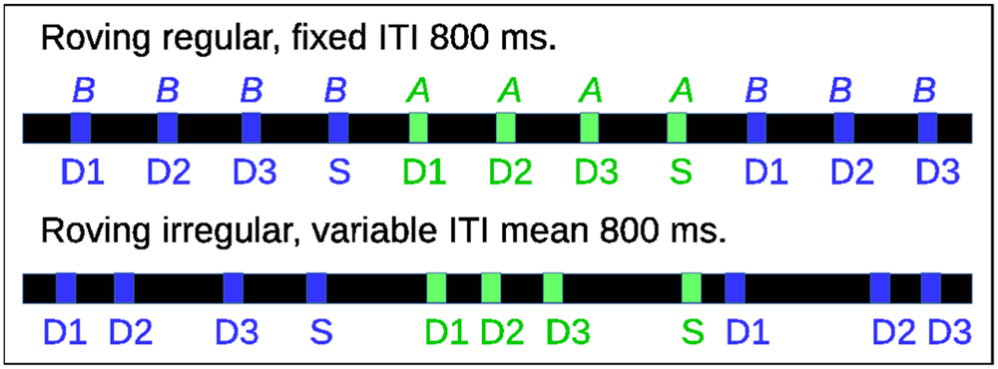
Schematic of roving sensory streams. Temporally regular and irregular roving sensory streams with *n* = 4. 50 ms stimuli *A* and *B* are separated by ITI periods of mean length 800 ms. In the irregular stream individual ITIs are chosen at random from the range 500–1100 ms in 100 ms steps. Successive presentations of a given stimulus type are labeled by their level of deviance.

Multiple studies show that a given stimulus produces different neuronal responses depending on whether the stimulus appears as implicitly expected according to the previous streaming^7,18,19,23–30,39,99,101,103–106^. To investigate this we employ a ‘roving’ sensory stream, (Fig. 4), often used in experimental studies. Again two stimuli, *A* and *B*, are used but each one is repeated several, ‘n’, times before switching to the other. The first stimulus after a switch is the most ‘deviant’ of that stimulus type, denoted ‘D1’, the second after a switch is the second most deviant, denoted ‘D2’, and so on. The final stimulus before a switch, ‘Dn’, is the ‘standard’, also denoted ‘S’. (We also sometimes use ‘D(n-1)’ and ‘D(n-2)’ as the standard (see below.)) Roving sensory streams switch between the two ‘single stimulus sensory streams’ which we denote *AA* and *BB*.

**Figure 5:**
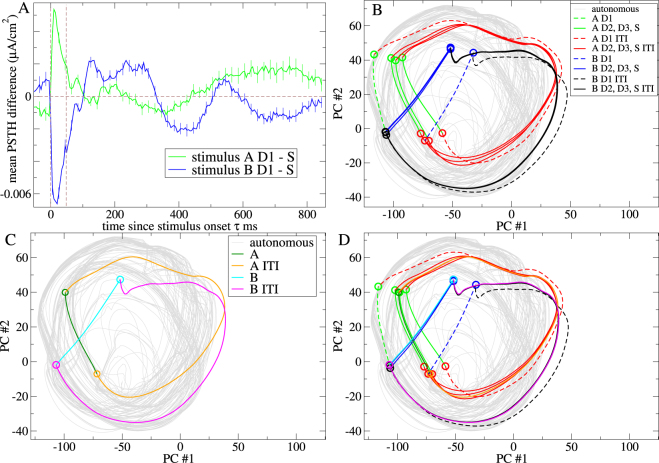
Network response to deviant stimuli presentations in the network simulation shown in Figs 1 and 2 under the *n* = 4 roving sensory stream with ITI 800 ms. (**A**) Differential dPSTH of neuroelectric activity for D1 presentations of both stimulus types (see key). Bars show SEM in the dPSTH. Vertical brown dotted lines show stimulus onset and offset. Simulation includes 10% noise. (**B**–**D**) Coloured line segments (see keys) show the trajectories of (**B**) the *n* = 4 roving sensory stream, (**C**) the *AA* and *BB* single stimulus sensory streams. Driven Lyapunov exponents *λ*_*D*_ are −0.0002 and −0.0027 respectively for these two streams. (**D**) The three sensory streams shown in (**B**) and (**C**) overlaid, projected onto the first two principal components of the autonomous activity. Lines show trajectory segments, circles show trajectory segment endpoints. The D1 stimuli and the ITI periods subsequent to them are shown dashed. Grey line shows the trajectory of the first two principal components of the autonomous activity. Simulations are deterministic.

We first illustrate the population activity. Figure 5(A) shows *differential* peri-stimulus time histograms (dPSTH) of the neuroelectric activity for the network simulation investigated above (Figs 1,2) under the *n* = 4 roving sensory stream with ITI 800 ms. dPSTH are generated by subtracting the activity generated by the standard S (D4) presentation of a given stimulus type, *A* or *B*, from the activity generated by deviant presentations of the same stimulus type (see Methods). Each stimulus type is shown separately. D1 stimuli show strong differential responses at stimulus presentation and modulations which persist throughout the ITI period.

Differential modulations are highly significant, as can be seen from the small error bars. This is because individual presentations of a given stimulus at a given deviance level are always preceded by identical sensory streams in this roving paradigm and because chaos is stabilized, *λ*_*D*_ < 0 < *λ*_*U*_. Given stimuli therefore generate distinct and reliable response at different deviancy levels. This is readily appreciated from the sensory stream driven activity projected in the principal components of the autonomous activity, Fig. 5(B).

**Figure 6:**
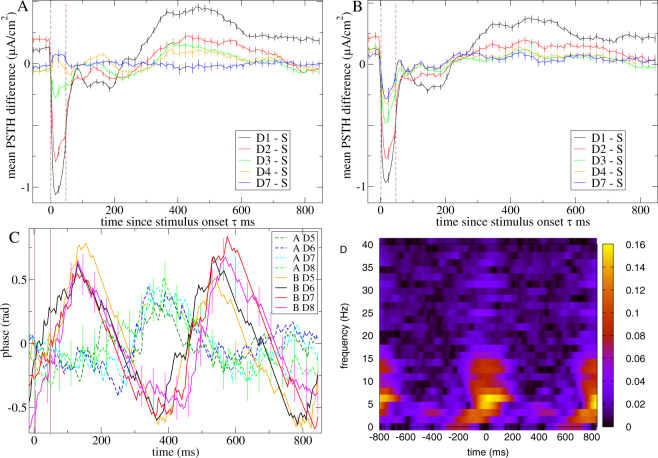
Stimulus locking of neuroelectric activity in a particular inhibitory network simulation which is only partially stabilized by the roving *n* = 12 sensory stream. (**A**,**B**) *Differential* dPSTH for one particular stimulus (stimulus *B*) at various levels of deviancy (see key) under (**A**) regularly and (**B**) irregularly timed streams. (**C**) PSTH of phase at the streaming frequency (see Methods) of the *induced* activity for several levels of deviancy (see key) and both stimuli. (**D**) Time-frequency plot of ITC (see Methods) in the *induced* activity with stimulus onset at zero averaged across all presentations, (except the first D1 and last D12), for stimulus *B*. (**A**–**D**) *λ*_*D*_ = 0.0004, *λ*_*U*_ = 0.001. Simulations include 10% noise. (**A**–**C**) Bars show SEM in the PSTH. Vertical brown lines show stimulus onset and offset.

**Figure 7:**
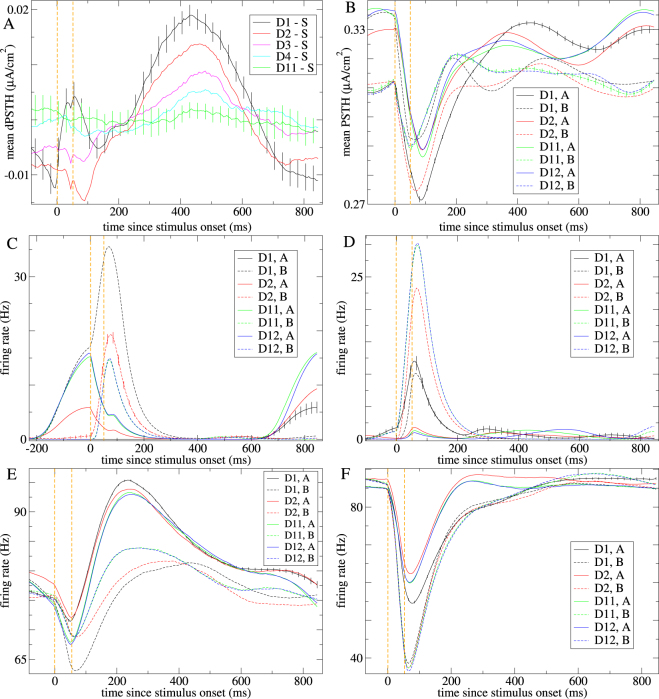
Examples of stimulus responses at different levels of deviancy (see keys) in an exemplar excitatory-inhibitory network simulation in which only half the excitatory and half the inhibitory units are variably driven by the sensory stream and which is only partially stabilized under the *n* = 12 roving sensory stream. (**A**) Differential neuroelectric activity dPSTH from inhibitory units not directly driven by the sensory stream for one stimulus type (stimulus *B*). (**B**) Mean neuroelectric activity PSTH from excitatory units directly driven by the sensory stream for both stimuli. (**C**–**F**) Firing rate PSTH for (**C**) an excitatory and (**D**,**E**) inhibitory units not directly driven by the sensory stream and (**F**) an inhibitory unit directly driven by the sensory stream. (**A**–**F**) Vertical orange dashed lines show stimulus onset and offset. *λ*_*U*_ = 0.0023, *λ*_*D*_ = 0.0006. Network connection probability 0.2. Relative excitatory to inhibitory input strength 0.74. Simulation includes 10% noise.

Oscillatory differential ITI period modulations, (Figs 6(A,B) and 7(A)) which are qualitatively as observed in population level studies^19,23–27^ occur because the low dimensional chaotic dynamics is dominated by low frequency oscillatory components. As illustrated in Fig. 5(B) D1 deviants produce responses which are relatively close to, but shifted away from the standards. This results in deviant and standard ITI period trajectories which rotate in a roughly concentric way generating the differential oscillations in the neuroelectric activity dPSTH, Fig. 5(A).

### Responses adapt slowly in the suppressed chaos regime

Experiments show that differential responses adapt gradually over surprisingly large numbers of stimulus presentations (e.g^19,25^.). Evidently as the number of repeat presentations of a given stimulus, D2, D3, …, increases, the activity adapts to the trajectories generated by the corresponding single stimulus sensory streams, *AA* and *BB*, shown in Fig. 5(C) and overlaid on the *n* = 4 roving stream in Fig. 5(D). In general if the *AA* or *BB* trajectory is stable the timescale of relaxation after D1 deviant stimuli is given by $|1/{\lambda }_{D}|$ where here *λ*_*D*_ refers to the driven Lyapunov exponent of the appropriate single stimulus trajectory. The slower decay to the less stable *AA* trajectory compared to the *BB* one is visible in Fig. 5(D).

**Figure 8:**
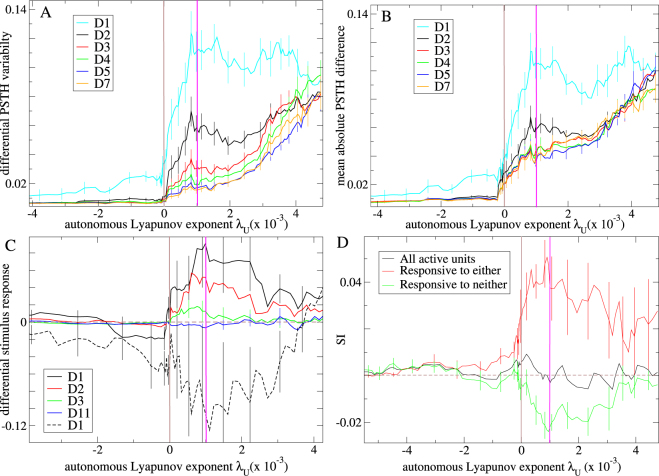
Large differential responses and slow adaptation in inhibitory networks under roving sensory streams. (**A**,**B**) Size of neuroelectric activity modulations, $\langle |{\rm{dPSTH}}|\rangle$, (|*x*| denotes absolute *x*) for various levels of deviancy (see key) averaged across the *whole 850 ms* stimulus and ITI period under (**A**) regularly and (**B**) irregularly timed sensory streams. 20 point moving averages. (**C**,**D**) Instantaneous responses averaged over only the *50 ms* stimulus presentations. (**C**) Expected size of differential instantaneous stimulus responses $({{\rm{dR}}}_{A}^{D1}+{{\rm{dR}}}_{B}^{D1})/2$ (solid) and $\surd {({{\rm{dR}}}_{A}^{D1}{{\rm{dR}}}_{B}^{D1})}_{\pm }$ (dashed) (see text). 15 point moving averages (sey key.) (**D**) Mean stimulus specific adaptation index, SI, calculated in three different ways (see Methods.) Black: all active units. Red: all units which respond phasically to either or both the D1 or S presentations. Green: all units which show no phasic response to either of the D1 or S presentations (see key.) (**A**–**C**) *n* = 12 roving sensory stream. (**D**) *n* = 4 roving sensory stream. (**A**–**D**) Results plotted versus the autonomous Lyapunov exponent *λ*_*U*_. Averages are over 112 network simulations calculated from *standardized* neuroelectric activity. Pink and brown lines show *λ*_*D*_ = 0 and *λ*_*U*_ = 0 as in Fig. 3(B). Simulations include 10% noise. Bars show SEM over the local moving averages (exemplar results only for figure clarity.) Both stimulus types *A* and *B* are averaged.

Furthermore due to the bundling of trajectories described above we find a similar gradual relaxation process occurs in the regime where chaos is only partially suppressed. Figure 6(A) shows neuroelectric activity dPSTH from a different purely inhibitory network simulation driven by the *n* = 12 roving sensory stream while Fig. 7(A) shows an example from a network including both excitatory and inhibitory units and in which only half of the units are directly driven by the sensory stream. In both cases driven activity is only partially stabilized, *λ*_*D*_ > 0, and adaptation occurs slowly over five or six stimulus presentations. These observations are summarized in Fig. 8(A) which shows the *absolute size* of differential modulations, $\langle |{\rm{dPSTH}}|\rangle$, (|..| denotes absolute value and 〈..〉 denotes average) averaged across the whole 850 ms stimulus and ITI period for various levels of deviancy in inhibitory networks driven by the *n* = 12 roving sensory stream. Only in the suppressed chaos regime do deviants generate large differential responses, peaking close to where chaos is just stabilized, *λ*_*D*_ = 0. The strongest dependence on repetition number is also shown in this regime.

The above example indicates the following explanation for these results. As *λ*_*U*_ increases responses at different levels of deviancy become more distinct and differential fluctuations (signal) larger. When *λ*_*D*_ increases past zero unreliability due to unsuppressed chaos (noise) increases. Because the dPSTH are calculated from standardized neuroelectric activity time series, $\langle |{\rm{dPSTH}}|\rangle$ reflect the relative size of signal to noise which decreases as the chaotic noise increases. Differential dPSTH depend on deviancy level throughout the suppressed chaos regime because stimulus response continues to reflect the preceding sensory stream even when chaos is only partially suppressed.

### Differential modulations decrease in magnitude when streaming is irregular

Differential responses are also known to depend on the temporal regularity of the adapting sensory stream^30^. Deviants in irregular sensory streams generate differential responses of smaller magnitude than their counterparts in regular streams. Here we investigate this using an irregularly timed roving sensory stream (see Fig. 4).

Differential responses to deviants under the irregularly timed stream (Fig. 6(B)) have similar form to their counterparts under the regularly timed stream (Fig. 6(A)) but are weaker and the error bars larger. This is confirmed in Fig. 8(B) which shows the magnitude of differential modulations, $\langle |{\rm{dPSTH}}|\rangle$, versus *λ*_*U*_ for inhibitory network simulations under the irregularly timed *n* = 12 roving sensory stream. The reduced size of the D1 differential response compared to Fig. 8(A) again occurs because the relative size of the signal component is reduced compared to noise. However in contrast to the case of temporally regular streaming described above where unsuppressed chaos was the sole source of unreliability, here unreliability is also caused by variability in the preceding streaming even when chaos is fully suppressed, *λ*_*D*_ < 0. In particular the increased variability of the S response (compare D7 in Fig. 8(A) and (B)) prevents adaptation after the third repetition, D3, and reduces the size of the D1 differential response. These observations are in qualitative agreement with the experimental studies (see Discussion.)

### Time-frequency responses

Multiple studies show that sensory streams phase entrain brain oscillations at the streaming frequency, and that this is weakened by temporal irregularity, for example^9–16,107^. Standard and deviant presentations of stimuli are also known to show characteristic responses^31,33,107,108^ and differences^19,35,109,110^ in their neuroelectric activity spectrograms. Stimulus presentations sometimes generate bursts of coherence in the theta and alpha bands associated with phase resetting of endogenous oscillations^35,108^. Here we investigate these issues in the *n* = 12 roving sensory stream. We employ two manipulations to remove the evoked stimulus response and reveal the underlying ‘induced’ activity.

The first ‘weaker’ manipulation is sometimes employed in experimental studies. From each time point *τ* ms after the onset of a given stimulus the mean value for that time point *τ* averaged across all presentations of that stimulus is subtracted (see Methods). We term this the ‘reduced’ activity. Supplemental Fig.S3(A) shows inter-trial phase coherence (ITC) across trials of a given deviancy level *D* of this reduced activity in a narrow band around the streaming frequency 1/0.85 Hz under the regularly and irregularly timed *n* = 12 roving streams versus the autonomous Lyapunov exponent *λ*_*U*_. Phase coherence peaks around the point where chaos is just stabilized for all levels of deviancy and is weaker when streaming is irregular. However it has a non-monotonic dependence on deviancy level *D*. This is because phase coherence is mainly due to the stimulus response which, due to response adaptation, is not fully removed by this manipulation. Indeed phase coherence at first decreases with increasing deviancy and then increases again, as expected if the *D*1 response is larger than the subtracted average and the *D*12 response smaller than the subtracted average. Phase coherence is however very low in stable networks demonstrating their absence of adaptation.

Supplemental Figs. S4 and S5 show time frequency plots of *excess* ITC and *excess* log power, respectively, in deviant *D*1 stimulus presentations compared to standard *D*11 stimulus presentations (see Methods) calculated from the reduced activity. Results are averaged across both stimuli and all network simulations with autonomous Lyapunov exponent falling within in prescribed ranges. Network simulations in the suppressed chaos regime, Supplemental Figs. S4(C), S5(C), show strong bursts of excess coherence and power locked to stimulus presentation, while these effects are weaker in more strongly chaotic and in stable networks (note different colour scales.) Such responses, which last several hundred milliseconds after stimulus offset and can extend up to around 80 Hz, are sometimes seen in experimental studies^19,31,33,35,107–110^.

Of more interest in the current study is a second ‘stronger’ manipulation. Here from each time point *τ* ms after the onset of a given stimulus of given deviancy level *D* we subtract the mean value for that time point *τ* averaged across all presentations of that stimulus of that deviancy level *D*. We term this the ‘induced’ activity. It reveals the underlying chaotic ‘noise fluctuations’. Figs. 6(C,D) illustrate this manipulation applied to the network simulation investigated in Fig. 6(A). PSTH of induced phase at the streaming frequency, Fig. 6(C), is highly similar across stimulus *B* presentations of different levels of deviancy, but less so for stimulus *A*. Phase for stimulus *B* appears to have twice the frequency of the sensory stream indicating higher mode locking for this particular network and stimulus. The phase PSTH for the two stimuli peak at different time points after stimulus offset, a common experimental observation. The time frequency plot of ITC, Fig. 6(D), averaged across all stimulus presentations (except the D1 and D12 stimuli, see Methods) for stimulus *B* shows a strong burst of ITC locked to stimulus onset around the theta to alpha frequency band (3–15 Hz), also often observed experimentally^19,31,33,35,107–110^.

**Figure 9:**
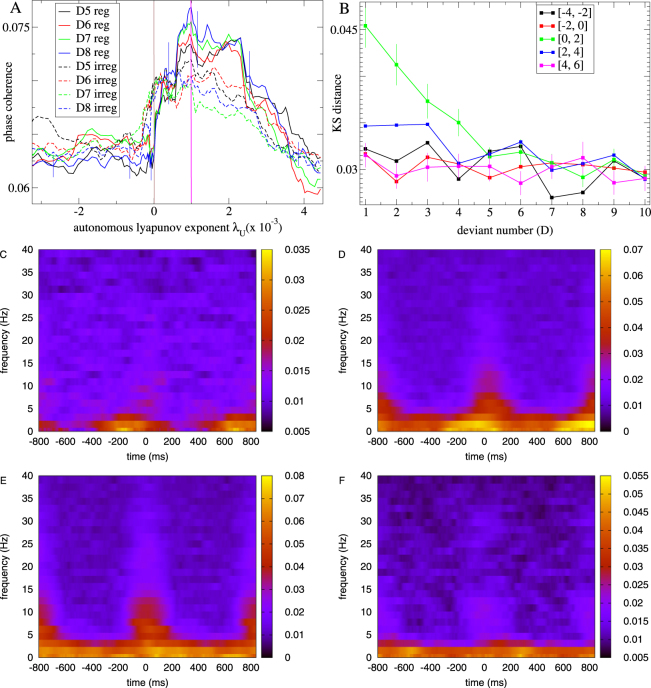
ITC (see Methods) across stimulus presentations of given deviancy level in the *induced* neuroelectric activity for the period between 300 and 600 ms after stimulus offset under the *n* = 12 roving sensory stream. (**A**) Phase coherence versus autonomous Lyapunov exponent for several deviancy levels in regular and irregular streams (see key.) 10 point moving average, bars show SEM in this average. (**B**) Distance between the phase distribution for stimuli of deviancy level D and that of standard D11 stimulus averaged across all simulations with autonomous Lyapunov exponent *λ*_*U*_ in the ranges [−4, −2], [−2, 0], [0, 2], [4, 6], (see key). Bars show SEM from the 300 and 600 ms averaging period. (**C**–**F**) Time frequency plots of ITC averaged across all stimulus presentations, except the first, D1, and last, D12, (see Methods) for simulations with autonomous Lyapunov exponent *λ*_*U*_ in the ranges (**C**) [−2, 0], (**D**) [0, 2], (**E**) [2, 4], (**F**) [4, 6]. (**A–E**) All results averaged across both stimuli *A* and *B*.

In Fig. 9(A) we investigate how ITC in the induced neuroelectric activity at the streaming frequency depends on the autonomous Lyapunov exponent *λ*_*U*_. In common with the reduced activity, Supplemental Fig. S3(A), this quantity peaks around the point where chaos is just stabilized and is weakened in irregular sensory streams. In contrast to the reduced activity it does not strongly depend on the level of deviancy. The fact that ITC in the induced activity does not strongly depend on deviancy does not mean that stimuli of different deviancy levels necessarily have similar phase PSTH profiles. Since phase PSTH can depend on the stimulus, as shown in Fig. 6(C), we expect it may take several stimulus presentations for the shift in phase locking to occur after the stimulus is switched in the roving sensory stream. To investigate this we calculate the distance between the phase distribution for deviancy level *D* and that for the standard *D*11 stimulus. This quantity is shown in Fig. 9(B) versus deviancy level *D* averaged across both stimuli and all network simulations within certain prescribed ranges of autonomous Lyapunov exponent *λ*_*U*_. It shows a gradual decay only in the suppressed chaos regime 0 < *λ*_*U*_ < 2, confirming the slow adaptation to a new phase locked state in the induced activity after a stimulus switch. This behaviour is also found in the reduced activity, Supplemental Fig. S3(B). Time frequency ITC Fig. 9(C–F) averaged across both stimuli and all network simulations within certain prescribed ranges of autonomous Lyapunov exponent *λ*_*U*_ show a strong burst of theta to alpha coherence at stimulus onset, especially in the suppressed chaos regime, Fig. 9(D).

Several studies show that low frequency brain oscillations can become phase entrained by sensory streams even when the streams are composed of multiple different stimuli^9,30,99–101,104^. This is possible in the current model because even streams composed of different stimuli can stabilize chaos by attractor switching as described above. To demonstrate this we apply the model to a simplified version of the task recently investigated by^9^. Participants discriminated the orientation of target visual stimuli embedded within streams of ‘random’ visual stimuli termed ‘distractors’ (see Supplemental Fig. S6(A)). The authors found phase entrainment was strong at the streaming frequency and increased with the temporal regularity of the sensory stream.

We investigate phase coherence of the ‘induced’ neuroelectric activity. To generate this the stimulus conditioned PSTH is subtracted from the neuroelectric activity for each different stimulus type separately, including targets and distractors, in the same way as described above (see Methods). We find phase coherence at the streaming frequency peaks (Supplemental Fig. S6(B)) close to the point where chaos is just stabilized at this ITI of 400 ms (compare Fig. 3(A)) when distractors are identical. A strong peak is also shown when distractors are random but similar to each other (clustered.) The peak is weakened by temporal irregularity. However the peak is absent when stimuli are totally random. Indeed while totally random stimuli streams can stabilize chaos (so that the trajectory of the driven network activity is independent of the initial network state at the onset of streaming) they do not phase synchronize the activity. The distractor stimuli used in experimental studies like^9^ should be considered ‘clustered’ since although different they have many features in common.

### Deviants generate larger instantaneous population responses than standards

We often find in inhibition dominated networks that stimulus presentations generate large negative instantaneous deflections in the neuroelectric activity. This can seen from the PSTH in the network simulation with both excitatory and inhibitory units, Fig. 7(B), as well as in the purely inhibitory network, Fig. 1(C). Here we investigate whether the magnitude of these instantaneous responses adapts with repetition number as often found in studies^18,28,47^. This effect is complicated by the possible presence of mode-locking. In networks where this occurs responses to odd numbered deviants (D1, D3, D5, …) may be larger than responses to even numbered deviants (D2, D4,…) for example. We also often find that D1 deviants generate instantaneous population responses of larger magnitude than standards for one stimulus type but not the other stimulus type in two stimuli roving sensory streams. The opposing character of the differential responses for the two stimulus types in the purely inhibitory network, Fig. 1(C), is apparent in the 50 ms stimulus presentation epoch in Fig. 5(A).

We measure the instantaneous stimulus response size, ${R}_{Z}^{D}$ to stimulus type *Z* = *A*,*B* of deviancy level *D* as the difference between the mean neuroelectric activity during the 50 ms stimulus presentation epoch and the 50 ms immediately preceding the stimulus presentation (see Methods). The differential response size for *D* stimuli is then defined as ${{\rm{dR}}}_{Z}^{D}=|{R}_{Z}^{D}|-|{R}_{Z}^{S}|$, where again |..| denotes absolute value and *S* = *D*12 is the standard stimulus. The stimulus averaged differential response, $({{\rm{dR}}}_{A}^{D}+{{\rm{dR}}}_{B}^{D})/2$, is shown in Fig. 8(C, solid lines) versus *λ*_*U*_ for inhibitory network simulations, while the opposing nature of the differential response sizes is illustrated by the quantity $\surd {({{\rm{dR}}}_{A}^{D}{{\rm{dR}}}_{B}^{D})}_{\pm }$, Fig. 8(C, dashed line). Here √(*x*)_±_ denotes sign (*x*)√(|*x*|). Throughout the suppressed chaos regime this latter quantity is large and negative for D1 stimuli. This effect is much weaker for later presentations after D1 (not shown). Although D1 differential responses sizes are often opposing in the two stimulus types, deviants generate larger magnitude responses in the neuroelectric activity than standards on average over both stimuli throughout the suppressed chaos regime, an effect which relaxes slowly over about four stimulus presentations, Fig. 8(C, solid lines). In fact both these effects can also be found in a simple phenomenological model where activity does not fully relax to an equilibrium state between stimulus presentations (see Supplemental). To show these effects the phenomenological model relaxation timescale parameter must be of the order of the time between stimulus presentations. In contrast here long timescales are created when network chaos is (partially) stabilized while network model timescale parameters do not exceed 50 ms and remain biologically reasonable. Thus the experimental observations of large excess instantaneous deflections in LFP^18,28,47^ for deviant stimuli compared to standards are qualitatively reproduced (see Discussion) only in the suppressed chaos regime.

### Single units also show adaptation

Stimulus response adaptation is also know to occur in multi-unit^18,28^ and single unit^17,111^ activity in both excitatory and inhibitory cells^20,21^. Figure 7(C–F) shows examples of single unit PSTH for standards and deviants from the network simulation including excitatory and inhibitory units under the *n* = 12 roving sensory stream. As can be seen the dependence on repetition number can be quite complex, both in the initial stimulus response and in the later modulations during the ITI. Stimulus presentations can generate phasic facilitating or suppressing responses to one or other stimulus type or both, Fig. 7(C,D). Stimulus induced modulations of tonically active units are also seen, Fig. 7(E,F). Stimulus induced modulations occur in both excitatory Fig. 7(C) and inhibitory units Fig. 7(D–F) and in units not directly driven by the sensory stream Fig. 7(C–E) as well as those that are, Fig. 7(F). Notice that this network simulation is almost completely stablized and the D11 and D12 responses are very similar.

These phasic responses result from the stimulus presentation induced ‘kicks’ described above. When the stimuli are presented the trajectory transiently moves away from the chaotic attractor region and then returns. During this period some single units silent during ITI periods are briefly activated. These units can suppress the ones tonically active during the ITI period resulting in negative deflections like those shown in Fig. 7(E,F) which may also appear in the population neuroelectric activity, Fig. 7(B). According to the above results we expect deviants to produce larger single unit responses than standards for one stimulus type but not the other, while deviant responses will be on average larger. On the other hand due to the presence of phasic activations in some units and suppression of tonic activity in others this may not show up if firing rates during stimulus presentation are simply averaged across all units.

To confirm these observations we calculate stimulus specific adaptation indices (SI) for single units, as in^17,111^, (see Methods.) SI vary from 1 to −1, depending on whether a unit is more active during the D1 or S presentation of a stimulus. The black line in Fig. 8(D) shows SI averaged across all the active units (see Methods) for many inhibitory network simulations under the *n* = 4 roving sensory stream versus *λ*_*U*_. Both stimulus types, *A* and *B*, are combined. When calculated in this way SI are close to zero throughout the range of *λ*_*U*_ and do not show a strong dependence on it. However when the SI average is restricted to include only units which show positive phasic responses to either the D1 or S stimulus presentations (see Methods), or both, Fig. 8(D, red), SI is significantly positive but only in the suppressed chaos regime. When the calculation includes only active units which do not show positive phasic responses to either D1 or S stimulus presentations, Fig. 8(D, green), the opposite is true. Thus phasic units are more strongly activated on average by deviants than standards while tonic units are on average more strongly suppressed by deviants than standards.

## Discussion

We investigated how stimulus streams interact with spontaneously generated dynamics in neural networks without any explicit learning mechanisms. Sensory stream adaptation may be a general property common to many different brain areas^13,22^ embedded in multiple possible neural substrates on different spatial and temporal scales during various behavioural task settings. To illustrate how a simple mechanism can unify a range of observations we have necessarily sacrificed model specificity for model generality. The model is the simplest one may imagine; a random network of identical units, each one described by a single dynamical variable with a single timescale driven by completely random stimuli. Despite this its emergent behaviour is so complex that it should be applied to each experimental setting individually to understand the related results. It can therefore provide deep insight into the origin of many seemingly contradictory experimental results. It will be particularly relevant in music, language and birdsong however repetitive sensory patterns may be generated by the motion of the animal itself. In particular chaotic network oscillations may occur as part of central pattern generators.

Remarkably we showed that stimulus sequences produce realistic repetition effects on neural response even without synaptic modification. Deviant stimuli generate long lived differential oscillatory modulations such as mismatch negativity and repetition positivity in population activity^19,23–27^. They are automatic, independent of the behavioural task and of attention^27^. Quantitative comparisons in a model of this generality are not possible since the form of the modulations depends on the task and brain area under study and also varies across individuals. We found good qualitative agreement with studies (e.g^19,25^.) when chaos is (partially) stabilized by stimulus streams. For greater agreement with experiment in future work the delay between the time of the physical stimulus and the time the signal reaches cortex, not included in the present model, should be taken into account, as part of more detailed modeling of the pre-cortical sensory processing. We showed partial stabilization allowed stimuli to generate distinct and reliable deviancy-level dependent responses in periodic roving streams.

We also found that deviants generate strong differential responses in temporally irregular streams^30,112^ but the response magnitude was weaker, in striking agreement with^30^. We showed this was because PSTH variability reflects stream irregularity in (partially) stabilized networks. Actually the effects of different possible forms of sensory stream variability on this model are complex. In the future this model will be profitably applied case by case to understand neural response in sensory streaming experiments with interacting factors. For example when stimulus contrast and stimulus novelty interact (e.g^38^.), or where temporal and type expectations interact^99–101^.

We also investigated the stimulus response specifically. In inhibitory networks D1 deviants on average generated larger population responses in neuroelectric activity than standards in one stimulus type. This tendency was only weakly shown for later repetitions. The *average* neuroelectric response size over both stimulus types was also larger for deviants than standards as is often observed in LFP studies^18,28,47^. Single units showed complex repetition dependent modulations both in their initial stimulus induced phasic activations and for longer periods during the ITI which are intriguingly similar to those found in recent studies^20,21,47^ and thought to result from recurrent network inhibition. On average single units showed stronger phasic responses than standards in striking agreement with studies at the multi-unit^18,28^ and single unit^17,111^ level.

In roving sensory streams we found adaptation to the new single stimulus stream after a deviant can happen very slowly over multiple trials, in agreement with multiple studies (e.g^25^.). In contrast to models based on synaptic mechanisms^113–115^ where adaptation timescales reflect synaptic time constants here slow adaptation occurs because stabilization of chaos brings the driven dynamics close to critical *λ*_*D*_ ≈ 0. The resulting adaptation timescales are around 20 times longer than the 50 ms synaptic time constant. There are several observations which provide convincing support for this which can’t be easily reproduced with purely synaptic mechanisms. First it accounts for the dependence of adaptation on temporal regularity of stimulus presentation^30^. Second it accounts for the generation of multiple adaptation timescales and adaptation to whole stimulus sequences^29,111^. Third it accounts for the fact that deviant stimuli may produce stronger responses than unadapted frequency matched controls^19,28,40,41^. However we do not expect network adaptation to operate in isolation from synaptic effects. Rather we hope recognition of the important fact that networks can adapt in a biologically plausible way will provide exciting insight into recent studies of how recurrent local inhibition and synaptic modifications interact^20,21,42,43,47^

Our results are in good agreement with experiments that demonstrate that low frequency brain oscillations become phase locked to stimuli presentations in streaming perceptual discrimination tasks^9–11,14,15,106,116–118^. Phase locking occurs across a broad range of cortical areas^14^, while locking outside the streaming frequency band is mostly limited to primary sensory areas. Stimuli presentations are associated with increased ITC and power predominantly in the theta to alpha band^31,33,107,108^, while deviants also generate differential responses in roving paradigms^19,35,109,110^, in qualitative agreement with this model.

Ongoing brain oscillations are often thought to be emergent properties of network mediated population dynamics^54–56,119,120^ close to a phase transition^121,122^. Several experimental studies have convincingly demonstrated they may be chaotic^123,124^. Interestingly low dimensional chaos may be stronger during slow-wave sleep than REM or wakefulness^58,125,126^, consistent with our hypothesis of chaos suppression by sensory driving. Intriguingly several studies have also found direct evidence for unstable periodic orbits in neural dynamics^127–129^, which are related to phase entrainment of chaos, while other studies directly demonstrate phase synchronization^130^.

Recurrent networks provide a natural substrate for various forms of neural computation and sequence learning^48–52,131,132^. SORN networks^133^ utilize plasticity to modify network structure and are therefore based on completely different principles to the current model. Like other studies^64,65,89,134–136^ in various task settings we find optimal performance in a critical state or at the edge of chaos. However we also show that consistency with experiment does not require this. We found stream history dependence in a wide regime where chaos is partially suppressed. This is surprising given the inherent instability of chaos^79^ but other studies find that significantly chaotic dynamics is consistent with neural information processing^77^. In the partially stabilized regime driven trajectories remain ‘bundled’ together so that the separation between them tends to a constant finite level. This is characteristic of chaotic synchronization^75,76,85^ and phase synchronization^63^. Phase stabilized chaotic trajectories combine divergence (high signal) with reliability (low internal noise) and therefore facilitate stream adapted response. Furthermore we found that the amount of stabilization, while strongly dependent on stimulus presentation frequency^95^, was only weakly dependent on the temporal or type predictability of stimuli in the stream. This allowed stimulus response reliabilites to be strongly determined by the variability of the preceding sensory stream. Finally we demonstrated this property emerged because stimuli move the network state away from its low dimensional chaotic attractor region. Thus we expect the results presented here to generally apply to chaotic networks in parameter regions where they can be stabilized, irrespective of some details of network physiology and structure.

## Methods

### Network simulations

We use a rate network model,

1 $${\tau }_{g}(d{f}_{i}/dt)=-{f}_{i}(t)+\gamma {\sqrt{({I}_{i}(t)-{I}_{\theta })}}_{+}+\alpha {\eta }_{i}(t){f}_{i}(t)$$

where *f*_*i*_(*t*) are the firing rates for the *N* firing rate units *i*. *I*_*i*_(*t*) is the input current to unit *i* given by,

2 $${I}_{i}(t)={V}_{Z}\,{g}_{i}^{Z}+{V}_{K}\sum _{j}{k}_{ij}\,{f}_{j}(t)\mathrm{.}$$

This equation models the variations of synaptic neurotransmitters which control cell firing rates. The synaptic timescale is set as *τ*_*g*_ = 50 ms. The term $\gamma {\sqrt{(I(t)-{I}_{\theta })}}_{+}$, ($\sqrt{I(t)-{I}_{\theta }}$ for *I*(*t*) − *I*_*θ*_ > 0 and zero otherwise) is the dependence of firing rate on input current close to saddle-node on invariant circle bifurcation to firing from quiescence as occurs in ‘Type I’ cell models. *γ* = 0.09 is a fixed parameter. *V*_*Z*,*K*_ = 60.0,5.0 mV are fixed parameters. *I*_*θ*_ = 4.51 *μ* A/cm ^2^ is the current at firing threshold. These firing rate units may be used to represent single cells or alternatively local populations of cells, as in^69^ for example.

*k*_*ij*_ is the fixed network connection matrix. We investigate two network models (i) a 500 unit purely inhibitory network and (ii) a 1200 unit network with 500 inhibitory units and 700 excitatory units. Most simulations for statistical calculations are performed on the 500 unit network. However we also demonstrate that the larger network has the capacity to show similar behaviour (see Supplemental Methods for more details.)

${g}_{i}^{Z}$ is the driving excitation for unit *i* during sensory stimulus *Z*. Simulations generally include two sensory stimuli, denoted *A* and *B*. These stimuli are always applied to the network for 50 ms time periods separated by longer inter-trial-intervals (ITIs). During ITIs a different excitatory input, denoted *X*, is applied in place of the sensory stimuli. The input ${g}_{i}^{Z}$ is drawn randomly from the same given distribution for each unit *i* and each stimulus *Z* in a simulation, whether it be *A*, *B* or *X*, and fixed for the duration of the simulation. The parameters are set so that the average input current to each unit *i* is just above firing threshold *I*_*θ*_ (see Supplemental Methods for more details.) All units *i* in the network are continuously driven above firing threshold by the external driving excitation by all stimuli *Z*. Only network inhibition can cause them to become quiescent for periods.

For generality we also investigate an excitatory-inhibitory network simulation where only half of the excitatory units and only half of the inhibitory units are directly driven by the sensory stimuli while the other half receive constant unvarying excitatory driving input. In this case the sensory stimuli *A*, *B* and background input *X* are determined as above but then ${g}_{i}^{A}$ and ${g}_{i}^{B}$ for half of the units *i* are set to ${g}_{i}^{X}$.

As a proxy for the LFP/EEG we use the absolute synaptic input current per unit $(1/N)|{V}_{K}{\sum }_{ij}{k}_{ij}{f}_{j}(t)|$ where |*x*| denotes the absolute value of *x* ^57,70^. In the simulation where only half of the units are directly driven by the sensory stream we divide this into four separate contributions, originating from the excitatory and inhibitory units directly and not directly driven by external sensory input.

In Eq. 1 *η*_*i*_(*t*) are iid Gaussian distributed noise terms with 〈*η*_*i*_(*t*)〉 = 0 and 〈*η*_*i*_(*t*_1_)*η*_*j*_(*t*_2_)〉 = *δ*_*ij*_ *δ*(*t*_1_ − *t*_2_). The parameter *α* controls the strength of this stochastic term and is always set to *α* = 0.1 so that fluctuations have the expected size 10% of the rates *f*_*i*_, except in Lyapunov exponent calculations and the time series examples where *α* is set to zero. The noise represents fluctuations arising from multiple sources including incoherent spike arrival times which have been averaged out in the present rate dynamics network model. Noise has no dynamical role in the results presented here; it is included purely to demonstrate their robustness.

All simulations of the noisy rate network were performed with a stochastic weak second order Runge-Kutta integrator^71^ while deterministic simulations are performed with a fourth order Runge-Kutta. All simulations are performed with integration time step 1 ms.

### Sensory stream experiments

Simulations of the *AB* and *AAABB* sensory streams were performed for 180 seconds after discarding a 30 second transient. Simulations of the roving sensory streams were performed until 120 repeats had occurred. This required 2390 second network simulations for *n* = 12 and 797 second network simulations for *n* = 4.

### Maximal Lyapunov exponent calculations

In this paper we make use of conditional maximal Lyapunov exponents^72–77^ which are calculated numerically. Their values depend on the temporally varying driving input as well as the network structure. In principle they also depend on the choice of initial conditions. All Lyapunov exponent calculations are made on the deterministic version of the model, Eq. 1, where the parameter *α* = 0, (see Supplemental Methods for more details of calculation).

### Principal component analysis

Principal components were calculated from the *N* × *N* covariance matrix obtained from the rate time series of all *N* units in a network simulation of length 180 seconds after discarding a 30 second transient. Principal components were calculated from autonomous network simulations constantly driven by the background input *X*. The grey trajectories in Figs 2 and 5 are time series of the autonomous network activity projected onto these first two principal component vectors. The coloured trajectory segments are the projections of the driven activity of the identical network simulation under the sensory streams onto the same first two principal component vectors of the autonomous activity. Sensory stream driven trajectories are divided into differently coloured segments according to the changes in driving input, *A* (green), ITI following *A* (driven by input *X*) (red), *B* (blue), ITI following *B* (driven by input *X*) (black). Segment endpoints are circled. Simulations under the sensory streams were performed until 88 presentations of each of the two stimulus types had occurred, resulting in 88 × 4 segments. This took approximately 150 seconds. All simulations were sampled at 5 ms intervals.

### Peri-stimulus time histograms

Stimulus and deviancy level conditioned peri-stimulus time histograms (PSTH) *H* ^*Z*,*D*^(*τ*) in the roving sensory streams are averages over the neuroelectric network activity, Fig. 7(B) or firing rate activity Fig. 7(C–F) time series sampled at 5 ms intervals at a particular time lag *τ* after the onset of the *D* ^*th*^ repetition of a particular stimulus *Z* = *A*,*B* for a particular network simulation. Differential dPSTH, Δ*H* ^*Z*,*D*^(*τ*) = *H* ^*Z*,*D*^(*τ*) − *H* ^*Z*,12^(*τ*), are shown in Figs 6(A,B) and 7(A) under the *n* = 12 roving sensory streams. Figure 5(A) shows differential dPSTH, Δ*H* ^*Z*,*D*^(*τ*) = *H* ^*Z*,*D*^(*τ*) − *H* ^*Z*,4^(*τ*) under the *n* = 4 roving sensory stream. Stimulus conditioned PSTH *H* ^*Z*^(*τ*) are calculated the same way except all deviancies *D* are included.

The mean *absolute* differential $\langle |{\rm{dPSTH}}|\rangle$ shown in Fig. 8(A,B) for each network simulation, stimulus *Z* and repetition D are given by $(850/5){\sum }_{\tau }|\Delta {H}^{Z,D}(\tau )|$ where |*x*| denotes the absolute value of *x*. dPSTH are calculated from standardized mean activity time series under the *n* = 12 roving sensory stream. The sum runs over all time lags *τ* from stimulus onset to the onset of the following stimulus.

Figure 8(C) shows results based on instantaneous neuroelectric activity responses *R* ^*Z*,*D*^ under the *n* = 12 roving sensory stream. ${R}^{Z,D}=(50/5){\sum }_{\tau }({H}^{Z,D}(\tau )-{H}^{Z,D}(-\tau ))$ where the sum over *τ* runs over only the 50 ms stimulus presentation epoch and −*τ* runs over the 50 ms time epoch immediately preceding stimulus presentation in 5 ms increments. Then differential instantaneous response is given by *dR* ^*Z*^ = |*R* ^*Z*,1^| − |*R* ^*Z*,12^|. Figure 8(C) shows (*dR* ^*A*^ + *dR* ^*B*^)/2 and ${\rm{sign}}(d{R}^{A}d{R}^{B})\sqrt{|d{R}^{A}d{R}^{B}|}$.

### Spectral analysis

First reduced and induced activity time series are created by subtracting the stimulus, *Z*, conditioned PSTH, *H* ^*Z*^(*τ*), and the stimulus *Z* and deviancy level *D* conditioned PSTH, *H* ^*Z*,*D*^(*τ*), respectively, from the *n* = 12 roving sensory stream neuroelectric activity time series. Irregular roving streams are treated the same way where PSTH are calculated from the same irregular neuroelectric activity.

To calculate time-frequency plots a Hanning window of length 640 ms is moved along the time series in 5 ms steps, *t*. From the complex coefficients *r*(*t*, *f*) (*e* ^*iθ*^(*t*, *f*)), log power, 2*log*(*r*(*t*, *f*)), and complex phase *e* ^*iθ*^(*t*, *f*) are computed and averaged to create stimulus onset locked power PSTH and inter-trial coherence (ITC). The absolute value of the mean complex phase is taken for the ITC which varies between zero and unity. PSTH are created in the same way as described above, for both stimuli *A* and *B* separately and all deviancy levels *D* separately. The time-frequency plot shown in Fig. 6(D) is the ITC averaged across stimulus *B* presentations of all deviancy levels except the first and last. These are omitted to avoid any end-effects originating from the stimulus switching in the 640 ms time window. The time-frequency ITC plots shown in Fig. 9(C–F) are created the same way except both stimuli and all network simulations within given ranges of *λ*_*U*_ are averaged. To create *excess* ITC and *excess* log power PSTH plots in Supplemental Fig. S4 and Supplemental Fig. S5 time-frequency spectrograms calculated from *D*11 stimuli of given type are subtracted from *D*1 stimuli of the same type. Then both stimuli and all network simulations within given ranges of *λ*_*U*_ are averaged. Again the *D*12 stimulus is not used to avoid possible effects originating from the stimulus switch.

To investigate phase coherence specifically at the streaming frequency in Figs 6(C) and 9(A,B), and Supplemental Fig. S3 a more frequency specific method is used. First the reduced or induced neuroelectric is divided into sections. Each section starts 480 ms after the offset of a *D*12 presentation of a given stimulus, say *A*, and ends 520 ms after the offset of the subsequent *D*12 presentation of the other stimulus type, say *B*. Thus each section includes presentations of only one stimulus type. Since the sampling period is 5 ms each section includes exactly 2048 grid points. In the irregular sensory stream 2048 grid point sections starting 480 ms after the offset of a *D*12 presentation are used. If a 2048 grid point section extends past the point of first presentation of the other stimulus type it is zero-padded from that point. Each section is band passed in a narrow band around the streaming frequency, (1/0.85)±0.3 Hz and the phase time series, *θ*(*t*), extracted using Hilbert transform. To calculate stimulus locked phase coherence, the distribution of phase, *p*(*θ*|*τ*, *Z*, *D*), *τ* ms after the onset of stimulus type *Z* of deviancy level *D* is obtained from multiple sections. For phase coherence we use the modulation index^137^ which is defined as 1 minus the normalized entropy of this phase distribution, $1-{\sum }_{i}p({\theta }_{i})\mathrm{log}\,p({\theta }_{i})/log(N)$ where *N* is the number of bins *i*. This quantity is averaged across the period 350 < *τ* < 650 ms and over both stimuli *Z* to obtain the phase coherence for each deviancy *D*. For the irregular stream we only include ITIs longer than 600 ms in this calculation. To calculate the difference between phase coherence for different deviancy levels in Fig. 9(B), Supplemental Fig. S3(B) the KS distance between the phase distributions is calculated. This is ${\sum }_{i}|P({\theta }_{i}|\tau ,Z,D)-P({\theta }_{i}|\tau ,Z,D11)|$ where *P* is the cumulative distribution, ∑ runs over the whole distribution and |..| denotes absolute value. This is averaged across 350 < *τ* < 650 ms and over both stimuli *Z* to obtain *KS*(*D*).

Phase coherence at the streaming frequency in Supplemental Fig. S6 is calculated in a similar way. First ‘induced’ activity is obtained from the neuroelectric activity by subtracting the stimulus conditioned PSTH *H* ^*Z*^(*τ*), where stimulus *Z* refers to the targets *A* and *B* and each of the six distractors, a total of eight stimuli. Next sections of length 16384 grid points (with time period of 16384 × 5 ms) are extracted, band passed in a narrow band around the streaming frequency (1/0.45) ± 0.4 Hz and the phase time series generated by Hilbert transform. Next the distribution of phase, *p*(*θ*|*τ*, *Z*), *τ* ms after the onset of target stimulus type *Z* = *A*,*B* is obtained from multiple target stimulus presentations. Finally the modulation index is calculated from the distribution and averaged across the period 300 < *τ* < 440 ms after stimulus onset and over both targets *A*,*B*. Irregular sensory streams are treated exactly the same way because each target is always followed by a 400 ms ITI.

### Single unit phasic analysis

Each unit was defined as *active* for a particular epoch if its mean firing rate exceeded 10^−5^ Hz during the epoch, for example during the final 400 ms of the ITI period following a D1 stimulus of type *A*. This minimum firing rate could be varied without change in results however some minimum firing rate is necessary for the calculation of stimulus specific adaptation indices *SI* ^17^. These are defined by $S{I}_{i}^{Z}=(SE{R}_{i}(Z,D\mathrm{1)}-SE{R}_{i}(Z,S))/(SE{R}_{i}(Z,D\mathrm{1)}+SE{R}_{i}(Z,S))$ for a unit *i* and stimulus type *Z* = *A*,*B* where ‘stimulus epoch rates’ *SER*_*i*_(*Z*,*D*1) and *SER*_*i*_(*Z*,*S*) are the firing rates of unit *i* during the 50 ms stimulus presentation epoch for deviant D1 and standard S presentations of stimulus type *Z*. For a unit to have an $S{I}_{i}^{Z}$ it must be active in either stimulus presentation, i.e. *SER*_*i*_(*Z*, *D*1) > 10^−5^ or *SER*_*i*_(*Z*, *S*) > 10^−5^.

The late epoch rates, *LER*_*i*_(*Z*, *D*1) and *LER*_*i*_(*Z*, *S*) for cell *i* and stimulus type *Z* are defined as the mean firing rate during the final 400 ms of the ITI period following the D1 or S presentations of stimulus type *Z*. The associated stimulus responsivities, *SR*_*i*_(*Z*, *D*1) and *SR*_*i*_(*Z*, *S*), are defined by *SER*_*i*_(*Z*, *D*1)/*LER*_*i*_(*Z*, *D*1) and *SER*_*i*_(*Z*, *S*)/*LER*_*i*_(*Z*, *S*). Supplemental Fig. S7 shows *SR*_*i*_(*Z*, *D*1) versus *LER*_*i*_(*Z*, *D*1) and *SR*_*i*_(*Z*, *S*) versus *LER*_*i*_(*Z*, *S*) for all 500 units in both D1 and S presentations of both stimulus types *Z*, (a total of 2000 points), for the partially stabilized network simulation shown in Fig. 8(A) under the *n* = 4 sensory stream. Single units are divided mainly into two distinct clusters. The cluster with SR close to unity and LER around 20 Hz contains the units tonically (or intermittently) activated by backgound input *X* during ITI periods. The cluster with LER around 10^−3^ Hz and SR around 10^−3^ contains the units phasically activated by D1 presentations (black plusses) and S presentations (red crosses).

A unit *i* is denoted ‘phasically responsive’ for a particular stimulus type *Z* and presentation type, D1 or S, if the associated *SR*_*i*_ > 10 and *LER*_*i*_ > 10^−5^. These phasically responsive units are the ones in the upper right quadrant demarcated by the pink dashed lines in Supplemental Fig. S7.

## Electronic supplementary material

Supplemental Information
