# Supplementary material for: Sensory Stream Adaptation in Chaotic Networks
Source: Sci Rep. 2017 Dec 4;7:16844. doi: 10.1038/s41598-017-16478-z (PMC5715003; doi:10.1038/s41598-017-16478-z)
Supplement: Supplementary file 1 — Supplemental Information [file 41598_2017_16478_MOESM1_ESM.pdf]

Sensory Stream Adaptation in Chaotic Networks, Adam Ponzi, Supplemental Information

Simple model of opposing responses

The origin of responses which are opposing in the two stimuli, as shown in Fig.5(a), but larger on average for deviants can be demonstrated with a simple phenomenological model. Possibly the simplest possible model of how stimuli affect neuroelectric activity, $F(t)$, can be described by,

$$\tau \frac{dF}{dt} = \gamma(t) - F(t) - \rho\delta(t)F(t)$$

where here τ is a timescale determined by the network dynamics (not the same as the timescale of the rate equation Eq.1), $\gamma(t)$ is noise reflecting the network activity and $\rho\delta(t)F(t)$ models the effect on $F(t)$ of the sensory stimulus where $\delta(t)$ is a spike and $0 < \rho < 1$ is a stimulus dependent strength. The stimulus is modeled as a spike only for simplicity, the discontinuity in derivative is not necessary. The sensory stimulus is coupled multiplicatively to the neuroelectric activity $F(t)$ so that its response depends on the current activity. Supposing sensory stimuli are streamed regularly separated by intervals of length T then the neuroelectric activity F_{i+1} at onset of the $i+1$ sensory stimulus is given by,

$$F_{i+1} = (1-\rho)\varepsilon F_i + \varepsilon B_i$$

where $\varepsilon = e^{-T/\tau}$ and $B_i = (1/\tau)\int_0^T \gamma(s+iT)e^{s/\tau}ds$. Then B_i are independent random variables so after many intervals i the expected value of F is

$$F^* = \frac{\gamma(1-\varepsilon)}{(1+\varepsilon(\rho-1))}$$

where γ is the expected value of $\gamma(t)$.

The size of the sensory response is ρF^*. Denoting the two stimuli as A and B in the roving sensory stream, the response to an A standard is $\rho_A F_A^*$, the response to an A deviant is $\rho_A F_B^*$, the response to B standard is $\rho_B F_B^*$ and the response to a B deviant is $\rho_B F_A^*$. Therefore,

$$dR(A)dR(B) = -\rho_A\rho_B(F_B^* - F_A^*)^2 < 0$$

and

$$dR(A) + dR(B) = (\rho_A - \rho_B)(F_B^* - F_A^*) > 0$$

The sum is positive because the equilibrium value F^* depends on the stimulus strength ρ and is inversely proportional to it. This is enhanced if τ is long, comparable to T, so that ε approaches unity.

Network Model Methods

In both fully inhibitory and inhibitory-excitatory models units are connected randomly with probability q. Connection strengths $k_{ij}^{I,E}$ for inhibitory I and excitatory E units are also random and drawn from a uniform distribution. Strengths $k^{I,E}$ are normalized by q with expectation $40/(qN_{I,E})$ and standard deviation $16\sqrt{1/12}/(qN_{I,E})$ where $N_I = 500$ is the quantity of inhibitory units and $N_E = 700$ is the quantity of excitatory units. Therefore when q is varied to generate network simulations with different dynamical properties the total strength of incomming (and outgoing) connections is fixed across different networks but its variance is not. Connections originating from excitatory units are positive, $k_{ij}^E > 0$, those from inhibitory units negative, $k_{ij}^I < 0$.

$\kappa_{inhib,excit}$ is a parameter which depends on whether cell i is inhibitory or excitatory. This allows us to vary the relative strength of excitatory input to inhibitory and excitatory cells. κ_{inhib} is fixed at unity for input to inhibitory cells. Many network simulations of different dynamical stabilities are produced by varying the connection probability q in both models and in the larger model by also varying the relative strength of driving to excitatory units, κ_{excit}. For the purely inhibitory model q is varied between 0.01 and 0.5. When q is smaller than about 0.2 network dynamics is chaotic, when larger than this network dynamics is stable (Supplemental Fig.S1). In the larger model κ_{excit} is varied between 0.1 and 1.3 for two values of connectivity $q = 0.15$ and $q = 0.2$. At these connectivities q when $\kappa_{excit} < 0.9$ network dynamics is inhibition dominated and chaotic, when κ_{excit} is larger than this dynamics is stable. q is also varied between 0.01 and 0.3 for fixed $\kappa_{excit} = 0.4$.

The input g_i^Z is given by $g_i^Z = \kappa_{inhib,excit}\sum_{j=1}^{10000} b_{ij}g_{ij}^Z$ representing a sum over 10000 inputs j for each unit i. The b_{ij} represent input synaptic strengths. They are drawn independently from a uniform distribution on $[0,2b]$ with expectation $b = 6\times10^{-4}$ for each unit i and input j. The g_{ij}^Z represent input firing rates. For each Z, i and j they are drawn independently from

a broad Pareto distribution $P_{\mu,\sigma}(x) = \mu\sigma/(1+\mu x)^{1+\sigma}$ with tail parameter σ and expectation $1/(\mu(\sigma-1))$. The parameter $\sigma = 1.75$ and μ is set so that the input rates g_{ij}^{Z} have expectation 20 Hz. The broad Pareto distribution is only used to ensure that different units receive a fairly broad distribution of total input currents after the 10000 inputs to each are summed. The distribution of driving inputs is not critical in this model. Any distribution which allowed each unit to have a different level of excitation would be sufficient. If this were not the case stimuli would be indistinguishable.

In the distractor task modeling[9] (Supplemental Fig.S6) six distractor sensory stimuli D were generated. For the simulations with completely random distractors, each distractor was generated in the same way as the target stimuli A and B described above. In the 'clustered' distractor case they were generated to be somewhat similar to each other as may be the case in experiments. First the length L of the vector $V_i = g_i^A - g_i^B$ between the two target stimuli driving inputs was calculated. Next the driving inputs for units i for a single distractor g_i^D were randomly chosen in the same way as described for stimuli A and B. Finally five more distractors were constructed by adding random vectors of length $L/20$ to g_i^D. To construct 'identical' distractors we use the same procedure but random vectors of length $L/10000$ are added to g_i^D.

Lyapunov Exponent Calculation Methods

Maximal Lyapunov exponents were calculated from time series of length 180 seconds after discarding a 30 second transient. Simulations were performed using a fourth order Runge-Kutta integrator with time step $\Delta t = 1$ ms. The maximal Lyapunov exponent λ was calculated by making a random perturbation to the trajectory to generate a second perturbed trajectory a distance $d_0 = 1\times 10^{-12}$ away from the original trajectory. The original and perturbed trajectories are iterated for one time step Δt. The original and perturbed systems were subject to identical temporally varying driving inputs. The separation $d_i(\Delta t)$ at each time step i is recorded and then a new perturbation of size d_0 is obtained by rescaling the vector between the perturbed and original trajectories. In this way the vector between the original and perturbed trajectories gradually orients to the maximally diverging direction. The first 5000 iterations after the perturbation were not used in the determination of the Lyapunov exponent to allow the perturbed trajectory to acquire the maximally diverging direction. Then λ is given by,

$$\lambda = \frac{1}{n\Delta t}\sum_{i}^{n} \ln\frac{d_i(\Delta t)}{d_0}$$

where $n = 150000 - 5000$.

Lyapunov exponents were observed to converge to final values within about 50000 iterations for the most unstable simulations and much faster for the more stable ones. Time series of Lyapunov exponents in Supplemental Fig.S8(a,b) show $(1/\Delta t)\ln(d_i(\Delta t)/d_0)$. The dependence of Lyapunov exponents on network parameters is described in Supplemental Fig.S1.

Since Lyapunov exponents can depend on initial conditions to calculate the autonomous Lyapunov exponent λ_U which best 'corresponds' to the AB sensory stream driven network simulations we calculate the Lyapunov exponent of the very slowly (adiabatically) driven network simulation under the alternating stimulus type AB sensory stream with the exact same inputs A, B and X but with a very long fixed ITI period of 25600 ms and the same initial conditions as the corresponding sensory stream driven network simulations. In almost all cases multistability has been absent and this value has been identical (except for fluctuations arising from the finite time length calculation) to the Lyapunov exponent of the autonomous network constantly driven by the fixed background stimulus X however (see Supplemental Fig.S2).

Stabilization of chaos by attractor switching.

Stabilization of chaos seems to result from switching between different stimulus dependent attractors. Supplemental Fig. S8(a,b) shows time series of driven maximal Lyapunov exponents (black) and their long time averages (red) for the network simulation investigated in Fig.1 and Fig.2 of the main text. Supplemental Fig.S8(a) shows automonous activity, λ_U, constantly driven by ITI period input X. The long time average Lyapunov exponent is positive. Supplemental Fig.S8(b) shows Lyapunov exponent, λ_D, of the same network activity driven by the AB sensory stream where all ITIs are fixed at 800 ms and stimulus type alternates. Large negative deflections are shown in the Lypaunov exponent time series following stimulus onsets (vertical lines, green, A and blue, B.) The long time average Lyapunov exponent is now negative. Supplemental Fig.S8(c,d) show stimulus onset locked PSTH of the driven Lyapunov exponent λ_D averaged across both stimulus types in all 22 simulations with long time average λ_D between 0 and 0.003, Supplemental Fig.S8(c), and all 8 simulations with long time average λ_D between 0.004 and 0.006, Supplemental Fig.S8(d). Negative deflections can be seen at both stimulus onset and offset. They are much more significant in Supplemental Fig.S8(c) than Supplemental Fig.S8(d).

To illustrate the attractors further we investigate their principal component projections (see Methods.) Supplemental Fig.S8(e) shows explained variance versus principal component number and Supplemental Fig.S8(f) shows network activity trajectories when the same network investigated in Supplemental Fig.S8(a,b) and in Fig.1 and Fig.2 of the main text is *constantly* driven by ITI period input X (orange), the stimulus A input (green), and the stimulus B input (blue). In Supplemental Fig.S8(f) the three trajectories are projected onto the first two principal components of the network activity when it is being

constantly driven by stimulus X. As can be seen in this particular example stimulus inputs X and B generate chaotic attractors while stimulus A happens to generate a stable limit cycle. The *AB* sensory stream driven trajectory is formed from repeated switching between these three relatively low dimensional attractors generated by the three different inputs. Although the dynamics on the stimulus X attractor (orange) is chaotic the transient switching portions of the trajectory between the attractors are stable. Notice that even though both the background input X and the stimulus B input (blue) generate chaotic attractors, the effect of stimulus B onset on Lyapunov exponent shown in Supplemental Fig.S8(b, blue line) is to produce a negative deflection. If the stabilizing effect of these stimulus presentations is sufficient to overcome the divergence on the X attractor then the sensory stream driven trajectory will be stable overall.

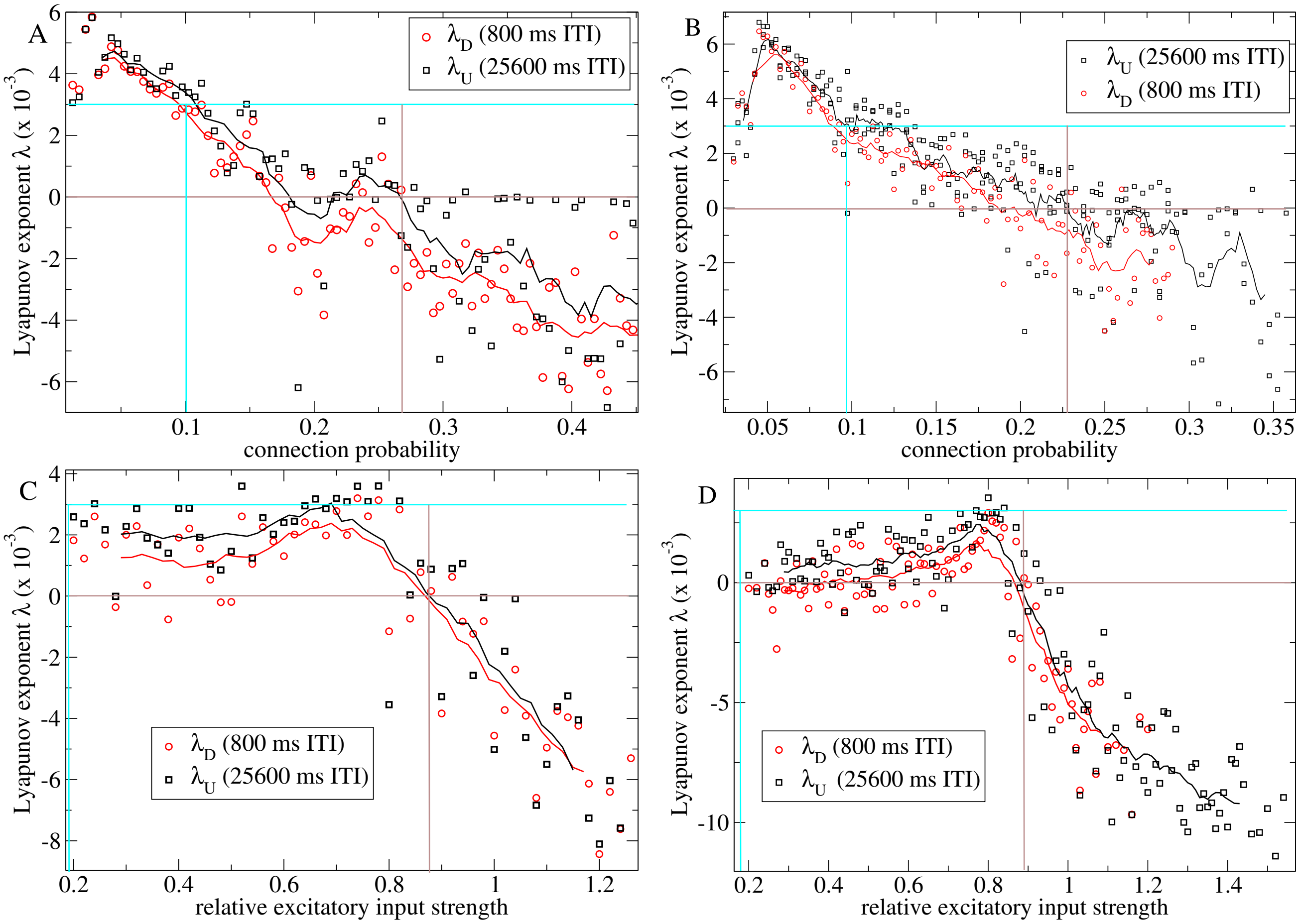

Figure S1. Dependence of Lyapunov exponents on network parameters. Black squares : λ_U 'autonomous' activity (i.e. driven by the *AB* sensory stream where stimulus type alternates and all ITIs are fixed at 25600 ms). Red circles : λ_D, driven by the *AB* sensory stream where stimulus type alternates and all ITIs are fixed at 800 ms. (a) Variation with network connection probability for 500 cell inhibitory networks. λ_U, 114 simulations. λ_D, 142 simulations. (Only simulations with connection probability < 0.45 are displayed in figure.) Lines show 10 point moving averages. (b) Variation with network connection probability for networks with 500 inhibitory cells and 700 excitatory cells. Relative excitatory input strength κ_{excit} is 0.4 of inhibitory input strength. λ_U, 236 simulations. λ_D, 105 simulations. Lines show 10 point moving averages. (c) Variation with relative excitatory input strength, κ_{excit}, for networks with 500 inhibitory cells and 700 excitatory cells and connection probability 0.15. λ_U, 53 simulations. λ_D, 54 simulations. Lines show 10 point moving averages. (d) Variation with relative excitatory input strength, κ_{excit}, for networks with 500 inhibitory cells and 700 excitatory cells and connection probability 0.2. λ_U, 128 simulations. λ_D, 93 simulations. Lines show 15 point moving averages. (a-d) All simulations are deterministic. The horizontal brown ($\lambda_U = 0$) and cyan ($\lambda_U = 0.003$) lines demarcate the regime where chaos is significantly suppressed by sensory streams with ITI around 800 ms. This regime roughly corresponds to the parameter values between the vertical cyan and brown lines.

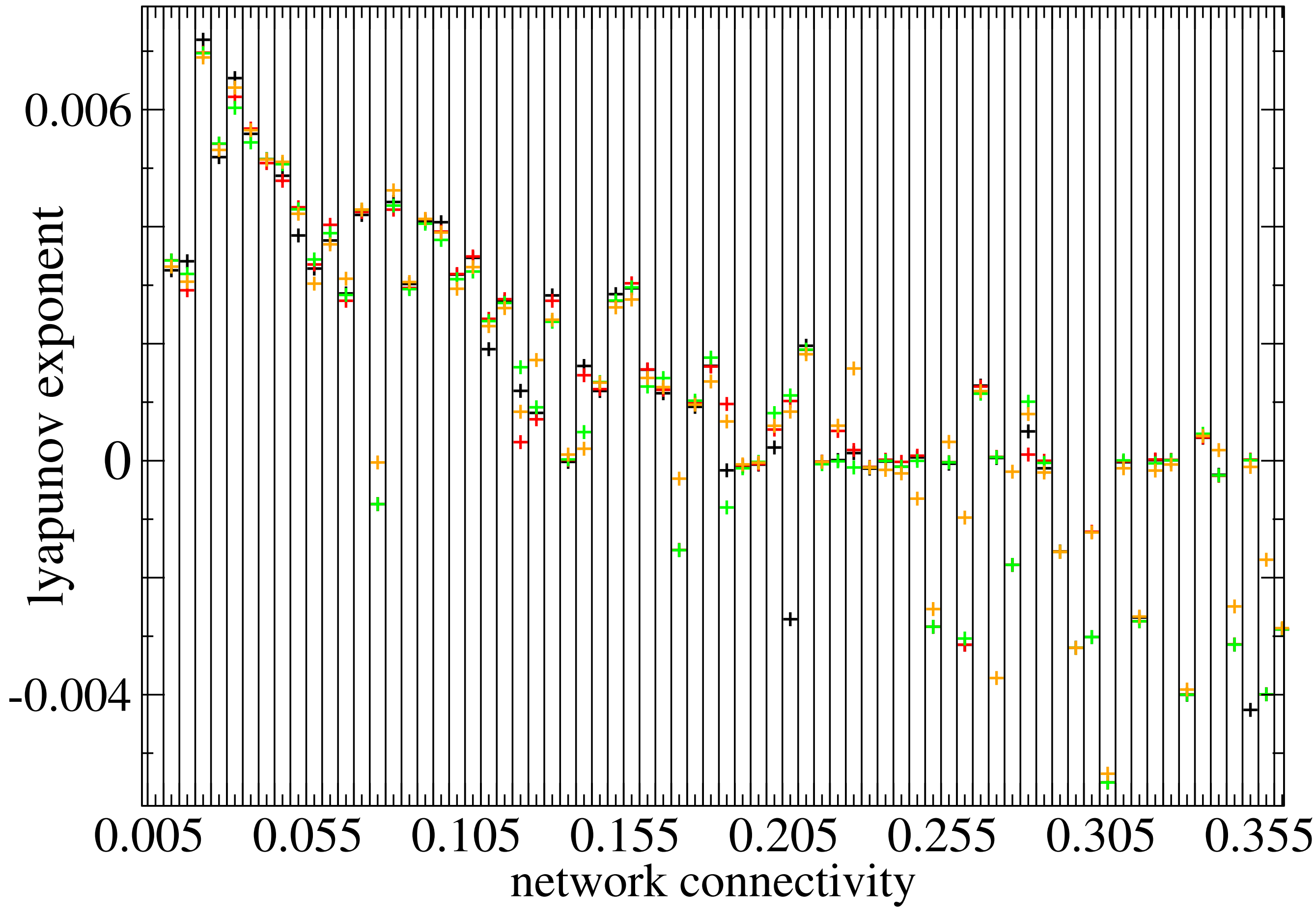

Figure S2. Lyapunov exponent λ_U versus network connectivity in 500 unit inhibitory networks. Each set of four symbols at each connectivity shows λ_U calculated from a particular network. Red, black and green symbols show λ_U calculated from 180 sec network simulations constantly driven by the background stimulus X for three different initial conditions. Multistability is evident in only a few simulations. Orange symbols show λ_U calculated *adiabatically* from 180 sec network simulations under the AB sensory stream where stimulus type alternates and all ITIs are fixed at 25600 ms for a single initial condition.

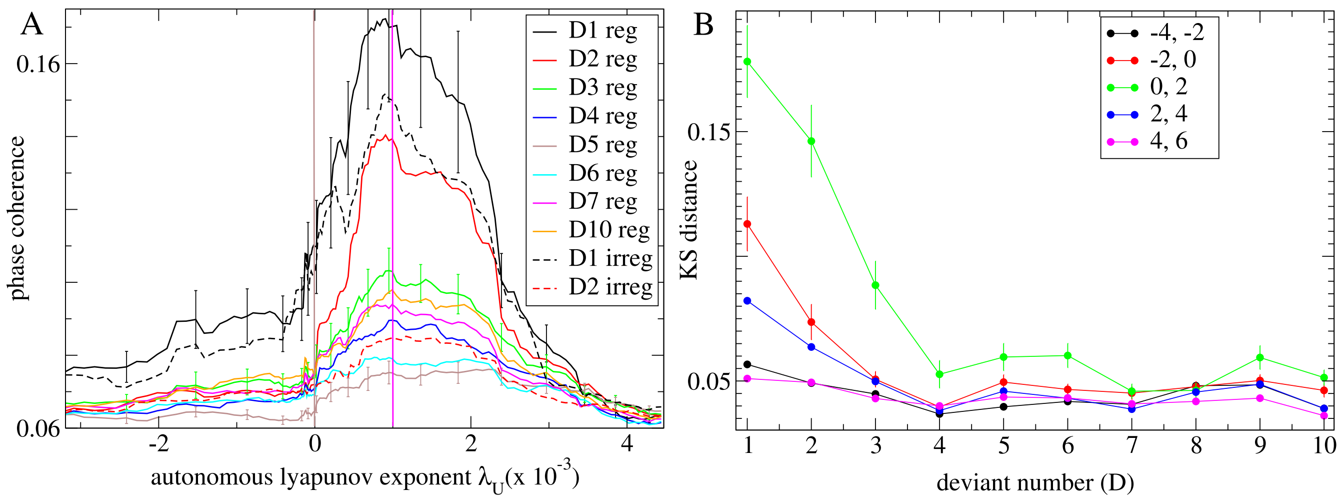

Figure S3. Phase coherence (ITC) at the streaming frequency (see Methods) across stimulus presentations of given deviancy level calculated from the *reduced* neuroelectric activity under $n = 12$ roving sensory stream. ITC is averaged across the period between 300 and 600 ms after stimulus offset. (a) Phase coherence versus autonomous Lyapunov exponent for several deviancy levels in regular and irregular streams (see key.) 10 point moving average. Bars show SEM in this moving average, exemplar results only for clarity. (b) Distance (see Methods) between the phase distribution for stimuli of deviancy level D and that of a 'standard' stimulus D11 averaged across all simulations with autonomous Lyapunov exponent λ_U in the ranges $[-4,-2]$, $[-2,0]$, $[0,2]$, $[4,6]$ (see key). Bars show SEM across the 300 to 600 ms averaging period.

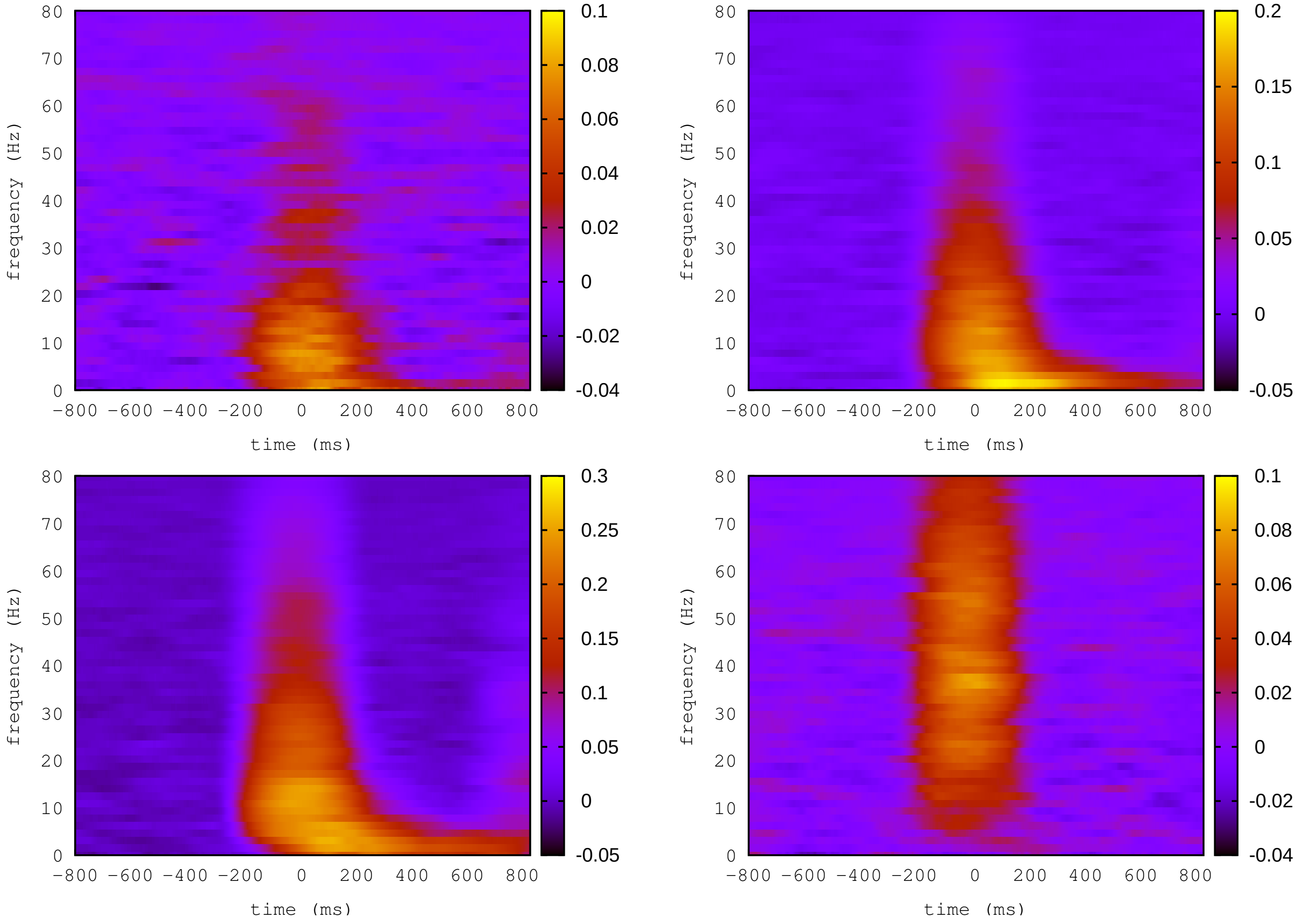

Figure S4. Time frequency plots of excess coherence in the 'reduced' activity in $D1$ deviants minus $D11$ standards (i.e. after 11 repetitions) under the $n = 12$ roving sensory stream averaged across all presentations of both stimuli A and B and all simulations (see Methods) with autonomous Lyapunov exponent in the ranges (top left) $-4 < \lambda_U < -2$, (top right) $-2 < \lambda_U < 0$, (bottom left) $0 < \lambda_U < 2$, (bottom right) $4 < \lambda_U < 6$. The time axis runs from -800 to 850 ms with 50 ms stimulus onset at 0.

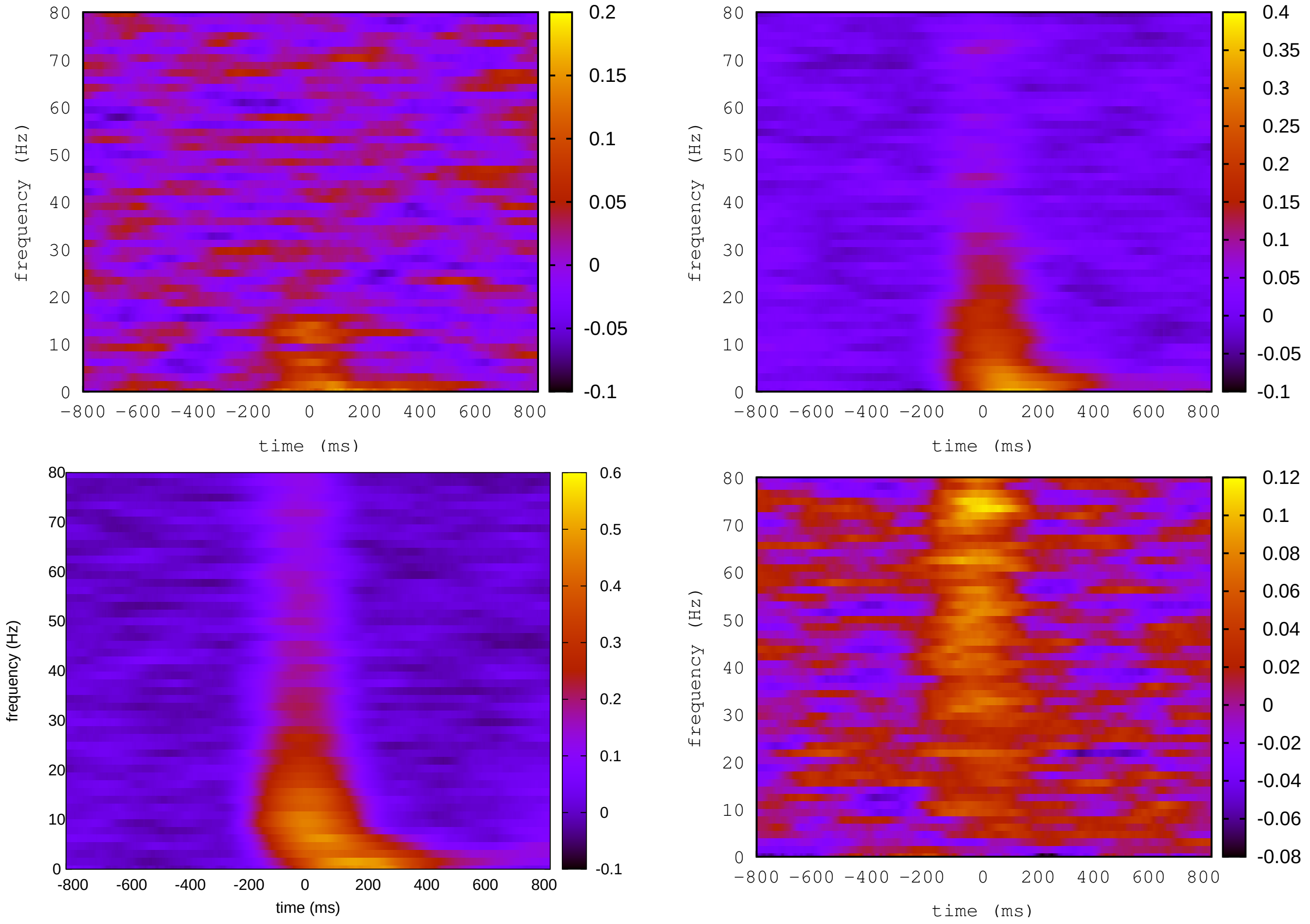

Figure S5. Time frequency plots of excess log power in the 'reduced' activity in $D1$ deviants minus $D11$ standards (i.e. after 11 repetitions) under the $n = 12$ roving sensory stream averaged across all presentations of both stimuli A and B and all simulations (see Methods) with autonomous Lyapunov exponent in the ranges (top left) $-4 < \lambda_U < -2$, (top right) $-2 < \lambda_U < 0$, (bottom left) $0 < \lambda_U < 2$, (bottom right) $4 < \lambda_U < 6$. The time axis runs from -800 to 850 ms with 50 ms stimulus onset at 0.

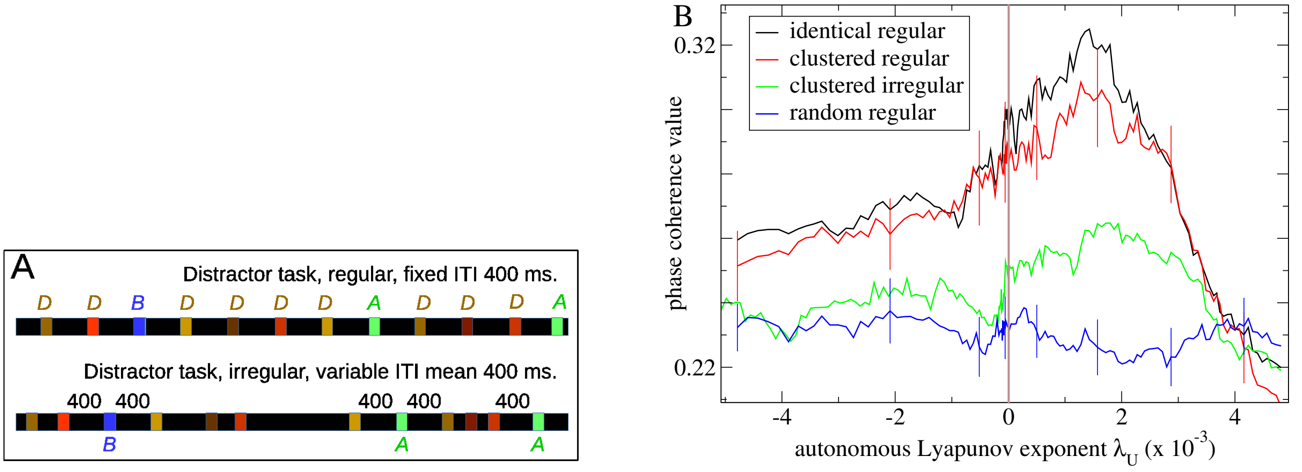

Figure S6. Temporal expectation in regularly and irregularly timed distractor streams. (a) Sensory stream used in the distractor task. 50 ms target stimuli, denoted by *A* and *B*, are separated by a random number of between 5 and 14 50 ms distractor stimuli, *D*. Each distractor presentation one from a set of six is chosen randomly. We use three different types of distractor sets : all six distractor stimuli identical, all six distractor stimuli completely random, or all six distractor stimuli random but similar, denoted 'clustered' (see Methods). In regular streams all ITIs between distractors and between distractors and targets were fixed at 400 ms. In irregular streams ITIs were drawn randomly from the five possible values between 200 ms and 600 ms in 100 ms steps, except for the ITIs immediately preceding and succeeding each target which were always 400 ms. Here in contrast to all other simulations the mean ITI is 400 ms, as in[9]. (b) Phase coherence of the 'induced' neuroelectric activity at the the streaming frequency, 1000/450 Hz, versus the autonomous Lyapunov exponent λ_U for multiple network simulations for different types of distractors and temporal regularity (see key.) Phase coherence is calculated in the window from 300 to 440 ms after target stimulus onset. 30 point moving averages across both target stimulus types. Bars show SEM in the averages (exemplar results only.) Simulations include 10% noise.

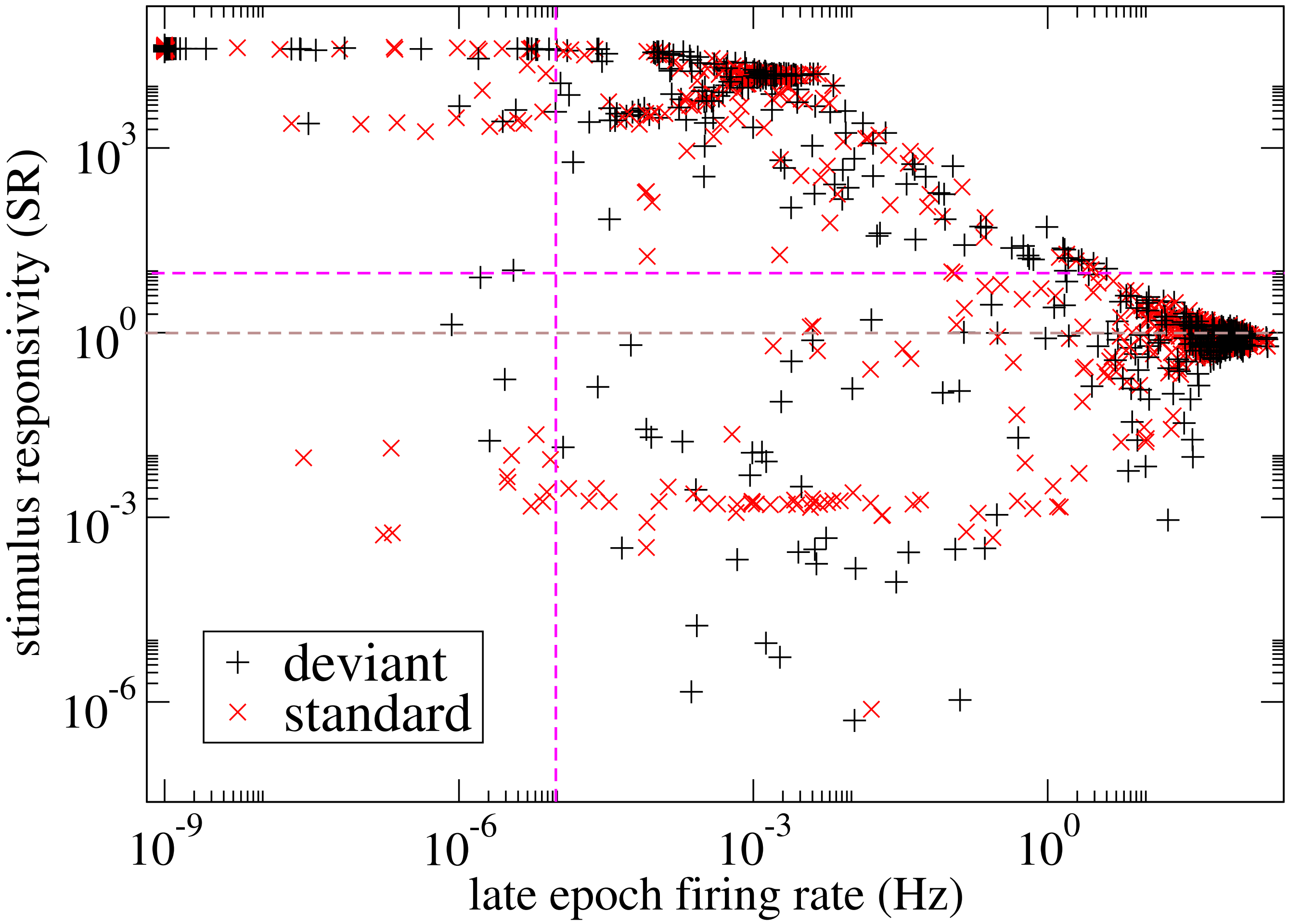

Figure S7. Mean SR versus LER for all 500 single units in the network simulation shown in Fig.8(a) under the $n = 4$ roving sensory stream. Results are shown for both D1 (red crosses) and S (black plusses) presentations of both stimulus types, resulting in 2000 points overall. The upper right quadrant demarcated by the pink dashed lines defines the phasically responsive region used in calculations. The brown dashed line shows SR= 1. 10^{-9} has been added to the LER of all units to facilitate plotting LER in log scale. The units with LER$< 10^{-9}$ form the cluster in the top left hand corner. Some units at very low SR are not shown in the figure. Simulation includes noise at 10%.

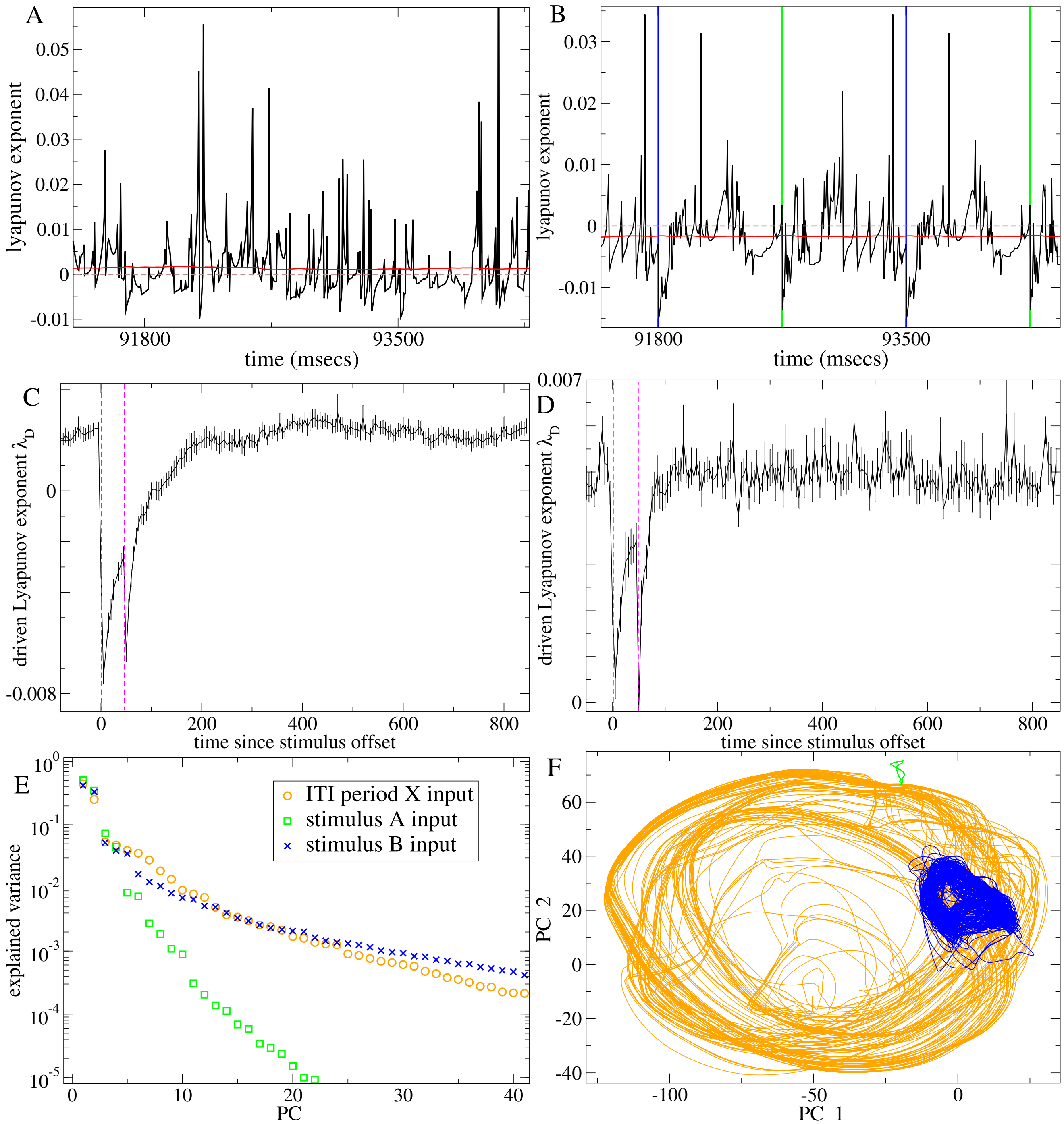

Figure S8. Stabilization of chaos results from switching between different stimulus dependent attractors (see Supplemental Text). (a,b) Time series of driven maximal Lyapunov exponents (black) and their long time averages (red) for the network simulation investigated in Fig.1 and Fig.2. (a) Automonous activity, λ_U, driven by input X. (b) Driven activity, λ_D, under the *AB* sensory stream where all ITIs are fixed at 800 ms and stimulus type alternates. Stimulus onsets are shown as vertical lines, green, *A* and blue, *B*. (c,d) Stimulus onset locked PSTH of driven Lyapunov exponent λ_D averaged across both stimulus types in (c) all 22 simulations with long time average λ_D between 0 and 0.003 and (d) all 8 simulations with long time average λ_D between 0.004 and 0.006. Bars show SEM in the averages across simulations. (e) Explained variance versus principal component number and (f) network activity trajectories when the same network investigated in (a,b) is *constantly* driven by ITI period input X (orange), stimulus A input (green), stimulus B input (blue). In (f) the three trajectories are projected onto the first two principal components of the network activity when it is being driven by stimulus X. (a-f) All simulations are deterministic with length 150 seconds.
